# Analysis of blood proteome in influenza-infected patients reveals new insights into the host response signatures distinguishing mild severe infections

**DOI:** 10.3389/fimmu.2025.1693728

**Published:** 2025-11-19

**Authors:** Klaus Schughart, Stephen C. Threlkeld, Subhashini A. Sellers, Willam A. Fischer, Jens Schreiber, Eva Lücke, Mark Heise, Amber M Smith

**Affiliations:** 1 Institute of Virology Münster, University of Münster, Münster, Germany; 2 Department of Microbiology, Immunology and Biochemistry, University of Tennessee Health Science Center, Memphis, TN, United States; 3 Baptist Memorial Hospital, Memphis, TN, United States; 4 Division of Pulmonary Diseases and Critical Care Medicine, Department of Medicine, University of North Carolina at Chapel Hill, Chapel Hill, NC, United States; 5 Clinic of Pneumology, Otto-von-Guerike University, Magdeburg, Germany; 6 Department of Genetics, University of North Carolina at Chapel Hill, Chapel Hill, NC, United States; 7 Department of Microbiology and Immunology, University of North Carolina at Chapel Hill, Chapel Hill, NC, United States; 8 Department of Pediatrics, University of Tennessee Health Science Center, Memphis, TN, United States

**Keywords:** influenza, proteome, differentially expressed proteins, transcriptome correlations, blood

## Abstract

**Introduction:**

Influenza infections result in a wide spectrum of disease outcomes, ranging from asymptomatic cases to fatal illness. While immunopathology contributes to an increased risk of hospitalization, the host factors that drive predisposition to ICU admission remain poorly understood.

**Methods:**

Here, we performed proteome analyses of sera from influenza virus-infected patients who were experiencing moderate disease without ICU admission or severe disease with ICU admission. A unique aspect of our study is that we monitored expression levels of more than 6,000 proteins whereas previous studies only analyzed a very limited number of protein markers.

**Results and Discussion:**

Comparing the responses in infected versus healthy individuals identified many differentially expressed proteins and related molecular pathways involved in lipid metabolism, iron metabolism, chromatin remodeling, and immune signaling in infected patients. These were amplified in patients with more severe disease, where immune signaling, proliferation/differentiation, and metabolic process pathways were increased. Our results suggest strong impacts of macrophage- and neutrophil-related responses. A unique aspect of our analysis is that it allowed us to relate the secreted host response in the blood (proteome) with stimulated responses in blood cells (transcriptome) in the same patients. Many differentially expressed proteins in the serum were not identified as differentially expressed genes in blood cells and therefore represent a not yet described set of biomarkers. Furthermore, we identified many strong correlations between blood cell transcriptomes and blood proteomes, which will allow us to validate or generate unique hypotheses of causal relationships between serum proteins and responses in blood cells during an influenza infection.

## Introduction

Seasonal influenza virus outbreaks result in substantial morbidity and mortality each year, posing a significant burden on public health. The clinical outcomes vary widely, ranging from no symptoms to life-threatening disease with several contributing factors, including the virus strain and the individual’s age, sex, genetics, and immune system status. In the most critical cases, death is often linked to an excessive immune response, marked by elevated activity of neutrophils, macrophages, and inflammatory cytokines. Mitigation of severe disease is challenging, and current antivirals that target viral proteins have limited efficacy once the disease has progressed to a state requiring hospitalization, where the focus is on supportive care to manage symptoms. Host-targeted therapies may pose an alternative strategy for these patients, where therapies targeting host factors that contribute to immunopathology may help abrogate progression to ICU admission. Proteomic approaches can pinpoint key virus-induced changes in host signaling pathways essential to disease progression and viral replication.

Prior studies of blood proteomes from influenza-infected adult patients identified differentially expressed proteins (DEPs) in infected versus healthy controls and patients with severe versus mild disease (1, 2). High levels of type II interferon (IFN-γ) and mediators of Th17 cell development were found in hospitalized patients with respiratory insufficiency (1). Increased plasma levels of several cytokines in patients with severe (critically ill) disease have also been observed (2). One limitation of these earlier studies was that they were performed using specific antibody-based Bio-Plex assays detecting only a limited number of selected markers (up to 27). However, performing analyses with methods that broadly detect a large number of proteins is important to more comprehensively understand the host response. More recently, we and others used the SOMAscan method, which detects a much larger number of proteins, to identify proteins in influenza-infected children from nasal washes/aspirates (3, 4). Additional studies have also investigated proteomes from nasopharyngeal and oropharyngeal swabs in children infected with different respiratory viruses (5, 6). These studies found some DEPs in infected versus healthy children. To our knowledge, similar studies have not been conducted in adults with influenza to identify large numbers of proteins differentially expressed in those with more severe disease.

Understanding how protein levels in the blood might be linked to gene transcription could also help improve diagnostics and predictability of disease progression. We and others have identified numerous cell transcripts of genes in blood cells that are differentially expressed in influenza-infected patients ( (7) and references therein). Many of these were related to antiviral, type I and II interferon responses, and chemokine/cytokine activation. Differentially-expressed genes (DEGs) increased in severe influenza disease compared to mild/moderate infections included chemokine/cytokine responses and neutrophil activation (7–10), suggesting possible overlap between the proteins and cell transcripts.

This study aimed to describe the host response to influenza infections in adults and relate it to intrinsic and external factors by identifying changes in blood protein levels of patients infected compared to healthy controls and by the severity of the disease. For this, we performed large-scale proteome analyses with plasma samples collected from healthy controls and influenza-infected patients with differing degrees of severity. We analyzed expression changes in 6,412 proteins, extending prior studies that monitored only a limited number of markers (fewer than 50). The resulting data were analyzed by bioinformatic approaches and related to previously identified transcriptome changes in blood cells. To our knowledge, this is the most comprehensive analysis of blood proteomes in influenza-infected patients.

## Materials and methods

### Patient cohorts

The patient cohort used for the analyses was described earlier (7). Briefly, patients with influenza infections and healthy controls were collected at five different sites. The total number of participants was 208, 81 were healthy controls, and 127 were influenza-infected patients, of whom 23 were admitted to the ICU. Healthy patients represented visitors to the hospital who volunteered to donate blood to our study. Samples from non-ICU infected patients were taken on the day of admission and were considered to have moderate disease. Samples from ICU patients were taken during their ICU stay (no specific time point). Additional six samples from ICU patients were taken at subsequent times during their stay. We selected a subgroup of 84 individuals that included 23 healthy controls and 61 influenza-infected patients. From three sites (Baptist Memorial Hospital (Memphis, TN USA), Otto-von-Gericke University (Magdeburg, Germany), University of North Carolina (Chapel Hill, NC USA). The selection of this subgroup was done manually to ensure equal representations for all groups where possible and to adhere to funding restrictions. Of the patients who reported to the hospitals with influenza, 26 were admitted to the ICU (**Table 1**). The number of females and males differed within groups. There were 21 females and 2 males in the healthy group (**Table 1**). In the influenza-infected groups, there were 32 females, 15 of whom were admitted to the ICU, and 29 males, 11 of whom were admitted to the ICU. The median age of the healthy controls was 44 years while the median age of the infected patients was 56 years (non-ICU: 60 years; ICU 46 years; **Table 1**). The full details of patient recruitment of the cohort have been described elsewhere (7).

**Table 1:** Demographics of cohorts.

Category	Healthy controls	Infected	Infected - non-ICU	Infected - ICU	P-values HC versus infected	P-values ICU versus non-ICU
Gender (males/females)	21F/2M	32F/29M			< 0.01	
*sum*	*23*	*61*				
Gender (males/females)			17F/18M	15F/11M		0.66
sum			35	26		
Age						
Age/years (median)	44	56	60	46	< 0.01	0.64
Age/years (range)	(24 -56)	(21 - 90)	(24 - 90)	(21 - 76)		
Age range 18-30	2F/0M	4F/1M	2F/0M	2F/1M		
Age range 30-65	19F/2M	20F/14M	10F/9M	10F/5M		
Age range > 65	0F/0M	8F/14M	5F/9M	3F/5M		

Number of participants stratified by gender, age, infection (infected: influenza infected, HC: healthy controls), and severity of disease (ICU: intensive care unit patients, non-ICU: patients not in intensive care). F: female, M: male. P-values were calculated by chi-square tests.

### Somascan proteome analyses

EDTA blood samples were collected from participants, cells were centrifuged, and supernatants and pellets were stored at -80°C until analysis. Plasma was centrifuged for 15 min at 2200 x g, and 60 μL of supernatant was used for the SOMAscan assay performed by SomaLogic, Boulder, CO as described previously (11–14). The SOMA panel used here contained aptamers for a total of 7,596 analytes/proteins. For some proteins, more than one aptamer was present, resulting in a total of 6,412 unique proteins that could be detected using this panel. Raw signals were then normalized as described (11, 12). The preprocessing steps included hybridization normalization, plate scaling and calibration, and the adaptive normalization by maximum likelihood (ANML), which normalized SomaScan EDTA plasma measurements to a healthy U.S. population reference, and values were then log_2_ transformed. Records with no gene symbol and duplicated gene symbols were removed (Data file: Normalized Somalogic proteome expression values, Data file: Descriptor Somalogic proteome expression). Of note, many non-secreted proteins were covered by the panel and were also detected in the blood of patients, most likely because some cell lysis occurred in infected lungs and during the preparation of the blood plasma.

### Mass spectrometry proteome analyses

For mass spectrometry analyses, blood samples were depleted of highly abundant blood proteins using the High Select Top14 Abundant Protein Depletion Mini Spin Columns (Thermo Scientific, catalog no A36369) as described by the manufacturer. Then the sample protein concentrations were determined using the Pierce™ BCA Protein Assay (Thermo Fisher Scientific, catalog no 23225). For mass spectrometry analysis, each sample contained 25 μg of protein in 150 µl of plasma depletion buffer (10 mM PBS) supplemented with 1% SDS and 100 mM ammonium bicarbonate, pH 8.1. The sample proteins were reduced with 6.25 mM DTT for 45 min at 50 °C, alkylated with 25 mM iodoacetamide for 20 min at RT in the dark, incubated with 20 mM DTT, and precipitated with 5 volumes of cold acetone. Proteins were sedimented at 16,000 xg at 4°C for 10 min. Protein pellets were washed with 100 µl of cold (-20°C) 90% acetone, air dried for 4 min, and re-dissolved in 50 µl of digestion buffer (100 mM HEPES, pH 8.3) containing 0.8 µg of Pierce Trypsin/Lys-C mixture (A40007, Thermo Fisher); the proteins were digested overnight at 37°C. A reference sample was generated by combining 2.5 µg aliquots of each digested sample. A set of 16 samples, each containing 22.5 µg of peptides in 45 µl of digestion buffer (100 mm HEPES pH 8.3), was labeled using a commercial TMTpro-16plex Mass Tag Labeling reagent kit (A44521, Thermo Fisher) according to the manufacturer’s protocol scaled to 45 µl. The total number of samples was analyzed in four separate runs. The four sets were labeled, and each set of labeled 16 samples included the same reference sample labeled with TMTpro-126 reagent. A set of labeled 16 samples was combined, vacuum dried, and reconstituted in 0.1% TFA at 0.3 µg/µl for further fractionation. 300 µl (90 µg) of the reconstituted mixture of labeled peptides was fractionated using Pierce High pH Reversed-Phase Peptide Fractionation kit (84868, Thermo Fisher) according to the manufacturer’s protocol - 8-step fractions (consecutively eluted with 10.0, 12.5, 15.0, 17.5, 20.0, 22.5, 25.0, and 50.0% acetonitrile) were collected. The collected peptide fractions were vacuum-dried and dissolved in 65 µl of loading buffer (3% acetonitrile with 0.1% TFA acid), and 5 µl aliquots were analyzed by LC-MS for peptide/protein identification and quantification. Acquisition of raw MS data was performed on an Orbitrap Fusion Lumos mass spectrometer (Thermo Fisher) operating in line with the Ultimate 3000RSLCnano UHPLS system (Thermo Fisher) using MS3 Synchronous Precursor Selection (SPS) method for TMTpro-16plex labeled samples with 160 min LC gradient. Post-acquisition analysis of raw mass spectrometry data was performed within a mass informatics platform Proteome Discoverer 2.4 (Thermo Fisher) using the Sequest HT search algorithm and human protein database (SwissProt, Homo sapiens, TaxID 9606, v.2022-10-12, 42315 entries). The reversed target database was used as a decoy database. The raw mass spectrometry data acquired for a set of 8 fractions (derived from the same mixture of labeled samples) were treated as ‘Fractions’ for post-acquisition analysis. Raw mass spectrometry data were then normalized as follows: the abundances of every peptide found in each sample were summed to determine total peptide abundance/amount. Normalization was performed by bringing the total peptide amounts in each sample to the same value by multiplication of individual peptide abundances of a given sample by the same factor specific to that sample. The resulting values were log_2_ transformed, missing values were set to zero, and then batch corrected for the runs using the function removeBatchEffect from the package limma [version 3.52.4, (15, 16)]. Proteins ALB, IGH, IGK, and IGL, which were depleted as described above, were set to a value of 1 (Data file: Normalized MassSpec proteome expression values, Data file: Data Descriptor MassSpec proteome expression). In total, 935 proteins were detected by this method. The analysis was performed at the Proteomics and Metabolomics Core (PMC) at UTHSC.

### Bioinformatic analyses of proteome data

Normalized Somalogic protein expression data were further analyzed using the R software (version 4.2.1 and 4.4.3, (17) and RStudio [version 2022.07.2 and 2024.12.1 (18)]. Multi-group comparisons and identification of differentially expressed proteins were performed with the package limma [version 3.52.4, (15, 16)] using the model design <- model.matrix(~ 0 + group); with group = healthy controls (HC) and infected patients (INF), or healthy controls (HC), infected patients not at ICU (non-ICU) and infected patients at ICU (ICU). Differentially expressed proteins were identified using the different contrasts from the model (INF versus HC, non-ICU versus HC, ICU versus HC, and ICU versus non-ICU) based on an adjusted *P* < 0.05 (Benjamini and Hochberg correction for multiple testing) and |log_2_| > 0.58 difference in expression levels. Volcano plots were generated with the package EnhancedVolcano, version 1.14.0 (19). Functional analyses of DEGs were performed using the R software package EnrichR (version 3.4, (20–22). For beeswarm graphs of expression levels, package beeswarm (23) (version 0.2.3.) was used. VENN diagrams were generated with the function vennPlot (http://faculty.ucr.edu/~tgirke/Documents/R_BioCond/My_R_Scripts/overLapper.R). For STRING network analysis, we used the STRING interactive website (https://string-db.org/cgi/input?sessionId=bZa9VJumLnb8&input_page_show_search=on), using basic settings: full STRING network, and evidence = true. Mass spectrometry normalized values were further analyzed using the R software (version 4.2.1 and 4.4.3, (17) and RStudio (version 2022.07.2 and 2024.12.1 (18)) with the packages and parameters described above.

### Analysis of correlations between proteome and gene expression data

Of the 78 patients used in the proteome study, 71 patients had previously been analyzed for gene expression in the blood by RNAseq (7). The remaining had insufficient RNA quality to perform transcriptomic analyses. For the 71 overlapping samples, we had proteome and transcriptome data from the same patients taken at the same time. We selected the proteome and transcriptome data from these patient samplings (Data file: Proteome overlapping samples and Data file: Transcriptome overlapping samples) and repeated the identification of DEPs and DEGs as described above. To determine correlations between the proteome and transcriptome, we combined all DEPs and all DEGs from the contrasts of non-ICU vs HC, ICU vs HC, and ICU vs non-ICU, and used Spearman correlation and BH (24) adjusted multiple testing *P* values to report significant results.

### Statistics

For the comparison of two groups, a two-way *t* test (numeric data) or chi-square test (categorical data) was used and performed in R. *P* < 0.05 was considered significant. Multiple testing adjusted *P* values were calculated according to Benjamini and Hochberg (24).

### Availability of data and materials

The original contributions presented in the study are publicly available. This data can be found here: https://doi.org/10.6084/m9.figshare.27826857 (Normalized Somalogic proteome expression values); https://doi.org/10.6084/m9.figshare.27827013 (Normalized MassSpec proteome expression values); https://doi.org/10.6084/m9.figshare.27852273 (Descriptor Somalogic proteome expression); https://doi.org/10.6084/m9.figshare.27852306 (Descriptor MassSpec proteome expression); https://doi.org/10.6084/m9.figshare.29882222.v1 (Proteome overlapping samples; and https://doi.org/10.6084/m9.figshare.29882255.v1 (Transcriptome overlapping samples). For additional data, see supplement tables. **Supplementary figures** are available as **Supplementary 11**.

## Results

### Blood proteomic signature in influenza-infected patients revealed unique DEPs

To identify the differentially expressed proteins (DEPs) in the blood of influenza-infected individuals, we performed proteomic analyses using a SOMAscan assay with samples from 61 influenza-infected patients and 23 healthy controls (Data file Normalized Somalogic proteome expression values, Data file Descriptor Somalogic proteome expression). Analysis of all proteins detected in the SomaLogic analysis by the GO-Cellular Component (GO-CC) ontology showed involvement of components from the extracellular matrix, vesicles, and granules, demonstrating that mainly secreted proteins were detected by this method.

**Figure 1:**
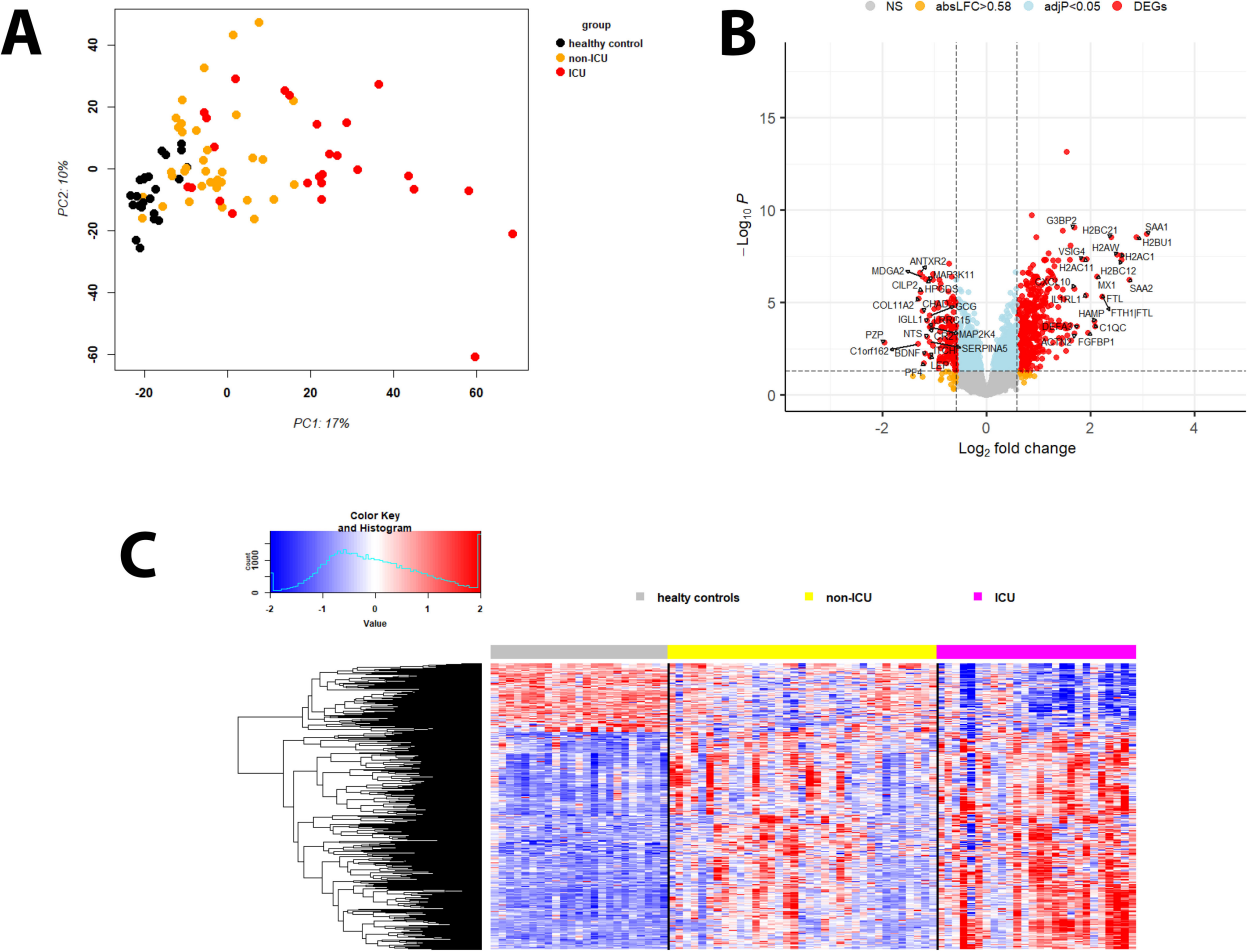
Principal component analysis and DEPs of infected versus healthy controls. **(A)** Principal Component Analysis (PCA) plot for protein expression values of healthy controls (black) and infected participants not in ICU (orange) and in ICU (red). **(B)** Volcano plot of DEPs for infected patients versus healthy controls. Y-axis: -log_10_ BH multiple testing adjusted *P* values, x-axis: log_2_ change. DEPs are colored red; the top 20 up- and downregulated (by log_2_ change) DEPs are labeled. Blue: not significant proteins with an adjusted *P* < 0.05. Orange: not significant proteins with an absolute log_2_ change > 1. **(C)** Heatmap of DEPs regulated in infected patients versus healthy controls for healthy controls (grey), patients not in ICU (yellow) and in ICU (magenta). Expression levels are scaled by row, red: higher relative levels, blue: lower relative expression levels.

A principal component analysis (PCA) of the proteins demonstrated separation between infected patients and healthy controls as well as between samples from infected patients who were in the ICU and those who were not in the ICU (**Figure 1A**).

An ANOVA analysis showed the strongest effect for infection status (PC1, p = 2.130693x10e-15) and smaller effects of sex, collection date, and age group (old: > 65 years; **Supplementary Table S1**). Analysis of interaction with infection status and age group was significant (*P* = 0.04), whereas interactions with sex and collection date were not. The effect of the collection site could not be analyzed because all healthy samples were collected at the Baptist Memorial Hospital. Virus type was not recorded.

Therefore, we only analyzed the contrasts for the different infection status (healthy controls, ICU, and non-ICU) and the influence of age on the responses for non-ICU and ICU patients (further below).

Comparing infected patients (both non-ICU and ICU cases) with healthy controls, 453 DEPs were upregulated while 143 were downregulated (**Figures 1B, C**: volcano plots showing the top 20 regulated DEPs and heatmap showing all DEPs; **Supplementary Table S2**). The top 10 upregulated DEPs included proteins involved in immune responses (SAA1, SAA2, H2BC21, MX1), iron transport (FTH1, FTL), and histones (H2BU1, H2AW, H2BC12, H2BC21; **Supplementary Table S3**). The top 10 down-regulated DEPs represented a more diverse group of proteins with functions in immune responses (PF4, HPGDS), differentiation/development/hormone activities (PZP, COL11A2, ANTXR2, CHAD), and neuronal functions (MDGA2, BDNF; **Supplementary Table S3**).

**Figure 2:**
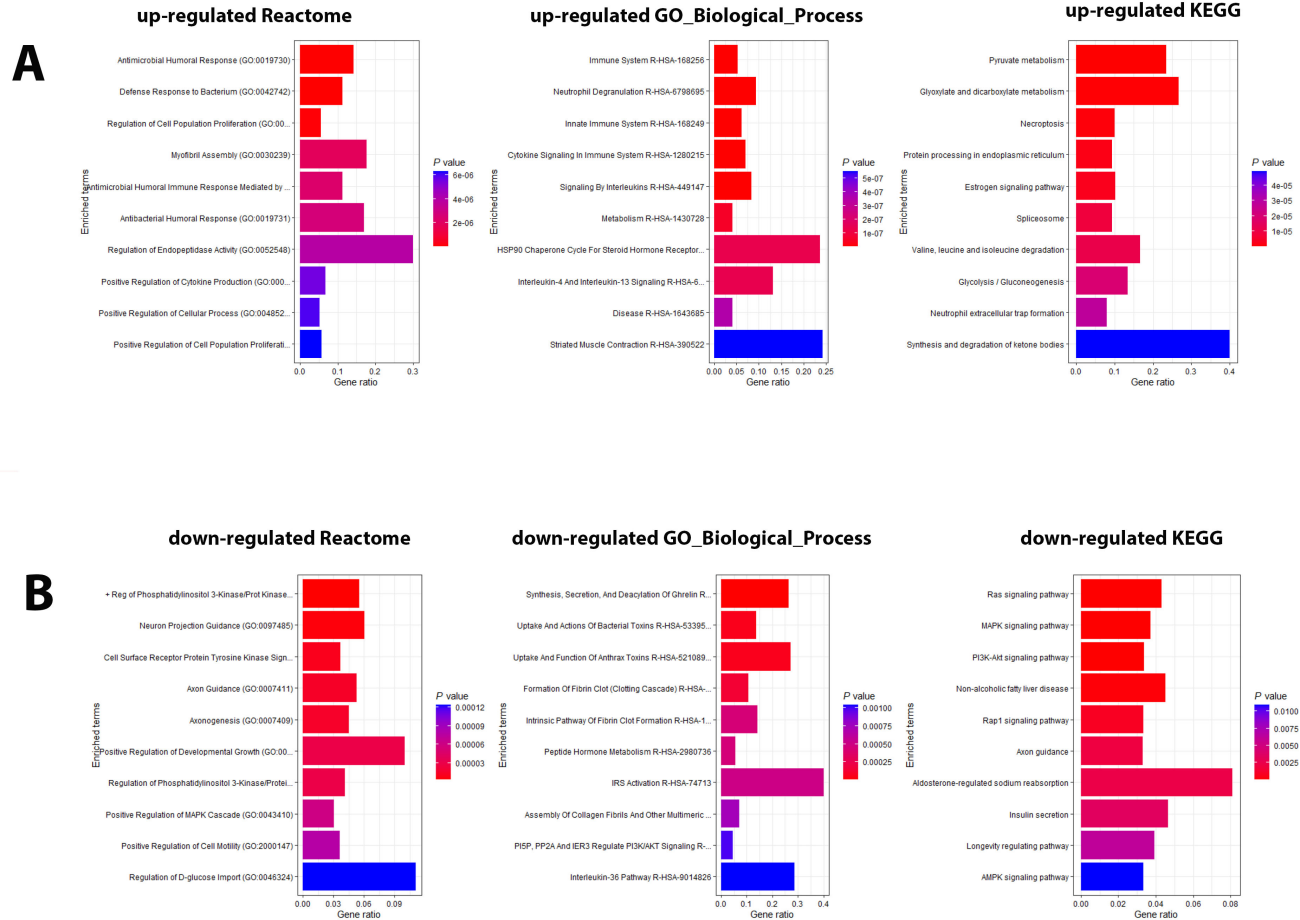
Pathway analysis of DEPs from the contrast of infected patients versus controls. **(A)** Functional analysis of up-regulated and **(B)** down-regulated DEPs from the contrast of infected patients versus healthy controls. Bars represent the top 10 (by *P* value) pathway hits of EnrichR analysis (Y-axis for gene ratios and color for *P* values) from databases "Reactome_2022", "GO_Biological_Process_2025", and "KEGG_2021_Human".

**Figure 3:**
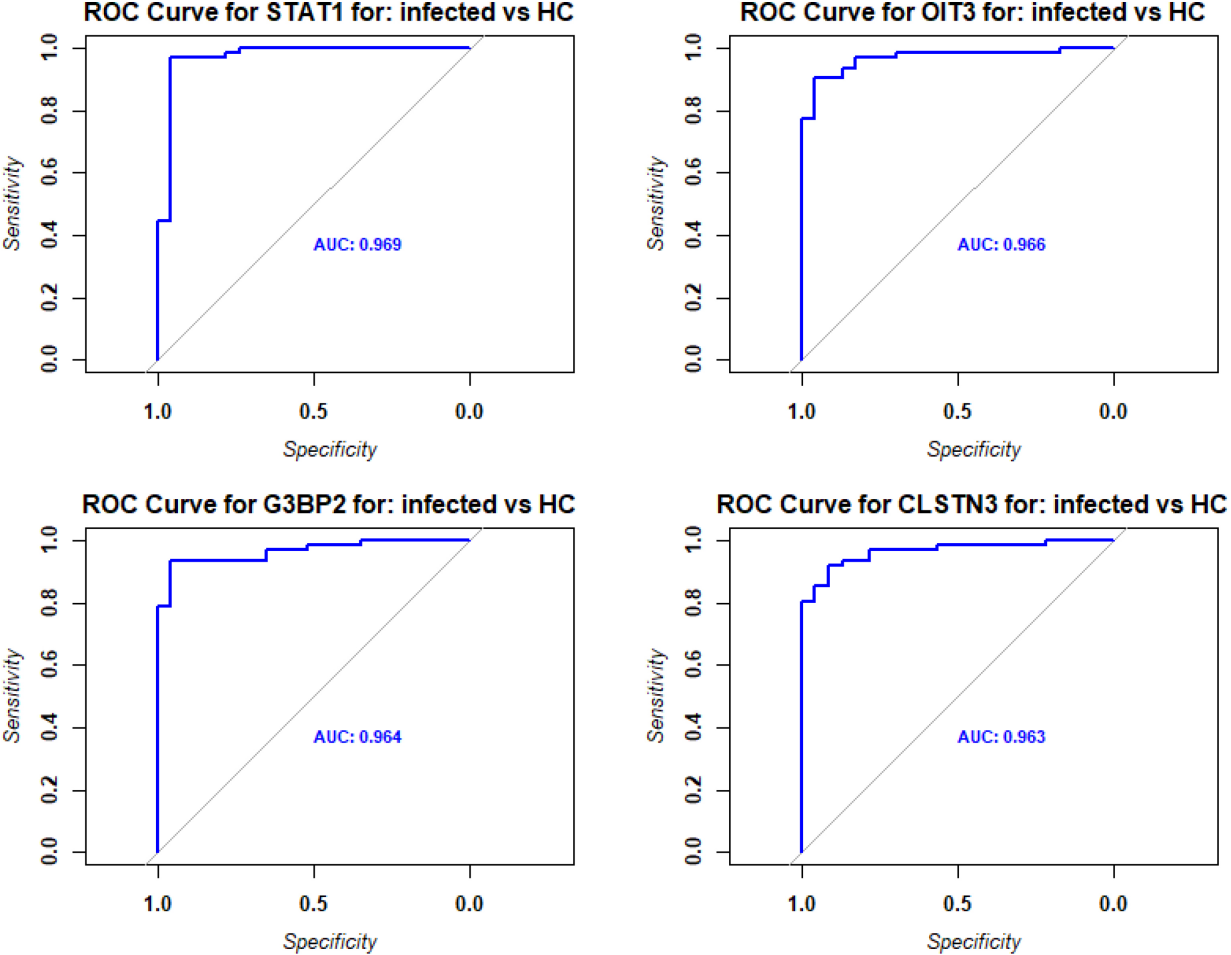
ROC analysis of DEPs from contrast of infected versus healthy controls. Receiver operating characteristic (ROC) curves (blue) for the top four DEPs with the highest predictive values (AUC: area under the curve).

Pathway analyses for the upregulated DEPs revealed responses that were mainly related to host immune defenses and metabolic pathways (**Figure 2A**). Downregulated DEPs response pathways were related to neuron guidance, response to toxins, and various signaling pathways (**Figure 2B**). The protein interactions of the top 200 DEPs (by absolute log_2_ change [LFC] using STRING network analyses) identified by the SOMAscan method showed prominent nodes for, *e.g.*, GAPDH, STAT3, CXCL10 (**Supplementary Figure S1A**), suggesting interplay between metabolic reprogramming, humoral immune response, and acute phase response limiting excessive inflammation and oxidative damage (25). STRING network analyses of the top 6 upregulated DEPs (SAA1/SAA2, H2BU1 (as only representative for H2B proteins), FTL, MX1, C1QC, and HAMP), revealed significant interactions with key proteins involved in inflammation and lipid metabolism, chromatin remodeling, interferon responses, iron metabolism, and complement (**Supplementary Figure S1B-G**), respectively, demonstrating that these DEPs are involved in key regulatory pathways of the host defense to pathogens. We then evaluated the predictive value of the DEPs by ROC analysis. Seventy-seven DEPs exhibited a very good AUC > 0.9 (**Supplementary Table S4**; **Figure 3** shows the top four DEPs with the highest predictive values).

### Validation of SOMAscan results by liquid chromatography mass spectrometry

Because many proteome studies use liquid chromatography mass spectrometry (LC-MS), we also performed LC-MS analyses for a subset of samples (9 healthy controls, 18 non-ICU patients, and 22 ICU patients). The PCA for expression levels obtained by LC-MS shows good separation between infected patients and healthy controls (**Supplementary Figure S2A**). The LC-MS studies detected fewer proteins compared to the SOMAscan analysis above (175 by LC-MS compared to 1314 for SOMAscan (combining DEPs for all three contrasts of non-ICU versus healthy controls, plus ICU versus healthy controls, plus ICU versus non-ICU; **Supplementary Figure S2B**, Data file Normalized MassSpec proteome expression values, Data file Descriptor MassSpec proteome expression **Supplementary Table S5**). About four times fewer DEPs were found to be significantly upregulated (118 by LC-MS versus 453 by SOMAscan for the contrast of infected versus healthy controls) or downregulated (36 by LC-MS versus 143 by SOMAscan; Volcano plot in **Supplementary Figure S2C** showing the top 20 up- and downregulated DEPs). This finding was most likely due to the lower number of samples in the LC-MS and the lower number of proteins detected in the LC-MS study (6,412 versus 935, respectively).

Of the 671 proteins that overlapped between the two assays, 365 were significantly correlated (*P* < 0.05) between SOMAscan and LC-MS. Of these, 184 were correlated with a coefficient > 0.6 and *P* < 0.05 (**Supplementary Table S6**; see **Supplementary Figure S2D** for examples). In conclusion, the LC-MS analysis confirmed our results obtained with the SOMAscan method. The remaining analyses below use only the results obtained from the SOMAscan assay.

### ICU admission is associated with shifts in genes involved in immune signaling, proliferation/differentiation, and metabolic processes

**Figure 4:**
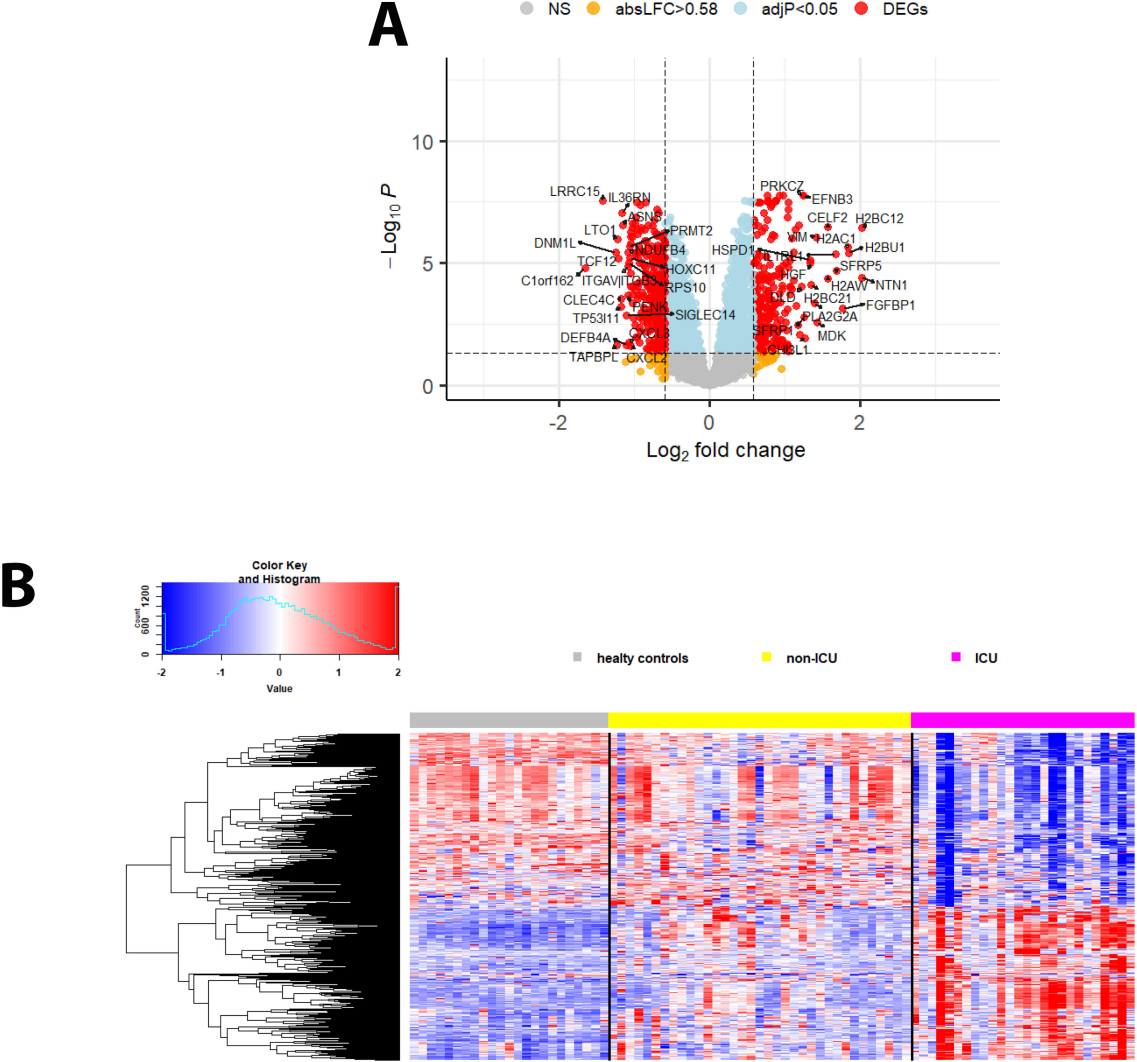
DEPs from the contrast of ICU versus non-ICU patients. **(A)** Volcano plot of DEPs for ICU versus non-ICU patients. ‘Upregulated’: higher in ICU, ‘downregulated’: higher in non-ICU patients. Y-axis: -log_10_ BH multiple testing adjusted *P* values, x-axis: log_2_ change. DEPs are colored red; the top 20 up- and downregulated (by log_2_ change) DEPs are labeled. Blue: not significant proteins with an adjusted *P* < 0.05. Orange: not significant proteins with an absolute log_2_ change > 1. **(B)** Heatmap of DEPs regulated in ICU versus non-ICU patients for healthy controls (grey), patients not in ICU (yellow) and in ICU (magenta). Expression levels are scaled by row, red: higher relative levels, blue: lower relative expression levels.

We then sought to identify the DEPs in the SOMAscan data between patients in the ICU and those who did not require ICU admission (non-ICU) by directly contrasting protein expression levels. The analyses identified 257 upregulated (higher in ICU) and 290 downregulated (higher in non-ICU; the volcano plot in **Figure 4A** shows the top 20 up- and downregulated DEPs; **Figure 4B** shows a heatmap of all DEPs; **Supplementary Table S2**). The top 10 DEPs expressed higher in ICU patients (**Supplementary Table S3**) included proteins involved in host immune response (IL1RL1, MDK), differentiation/proliferation (NTN1, FGFBP1, SFRP5), and DNA binding/histones (H2BC12, H2BU1, H2AC1, H2AW). The top 10 DEPs expressed higher in non-ICU patients (**Supplementary Table S3**) included proteins involved in host immune responses (LRRC15, TAPBPL, CLEC4C, IL36RN), negative cell proliferation/apoptosis (TP53I11, ASNS), and cell signaling/protein maturation (DNM1L, LTO1).

**Figure 5:**
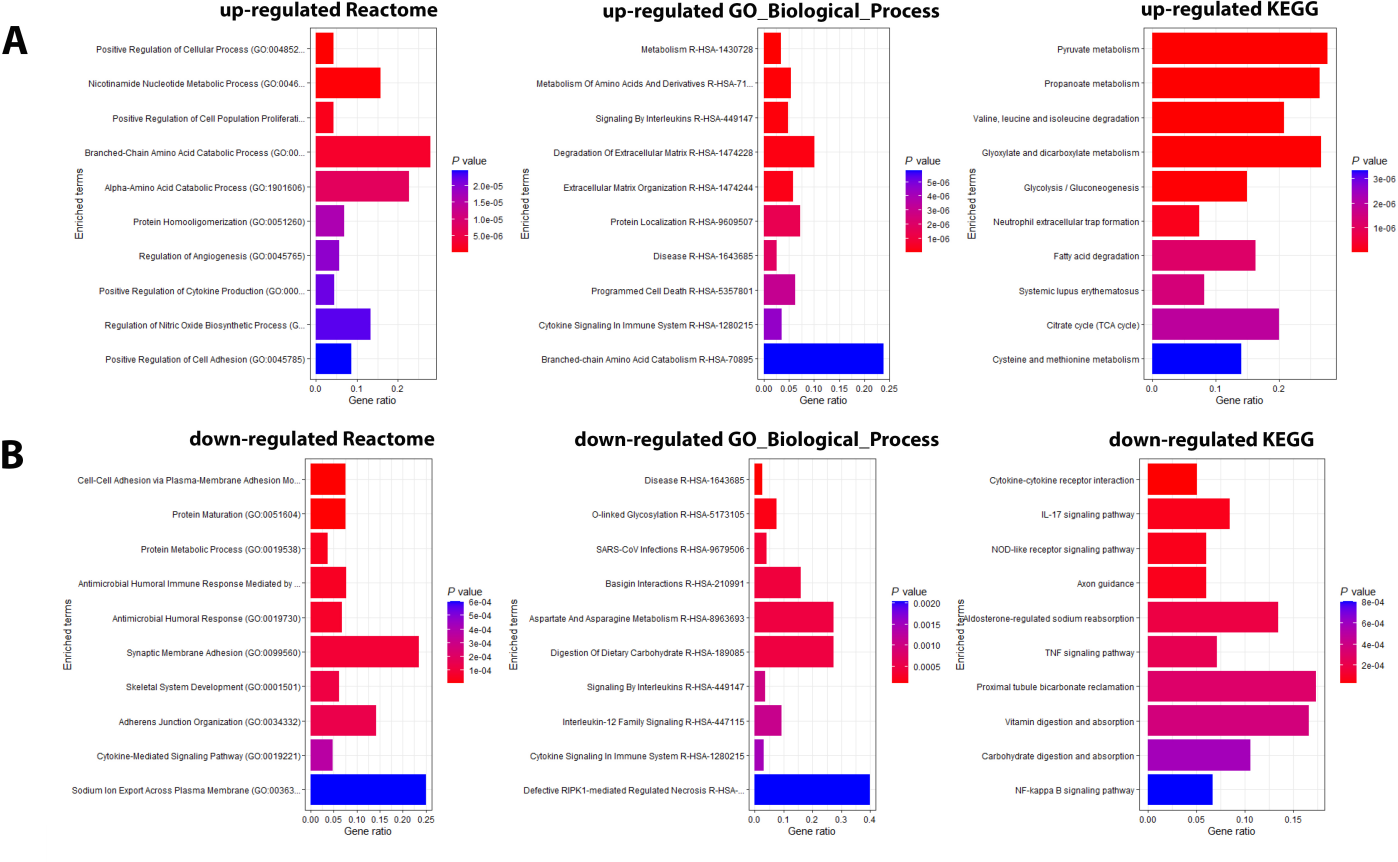
Pathway analysis of DEPs from the contrast of infected patients versus controls. **(A)** Functional analysis of up-regulated and (**B**) down-regulated DEPs from the contrast of ICU versus non-ICU patients. Bars represent the top 10 (by *P* value) pathway hits of EnrichR analysis (y-axis for gene ratios and color for *P* values) from databases "Reactome_2022", "GO_Biological_Process_2025", and "KEGG_2021_Human".

Pathway analyses for the DEPs higher in ICU patients (**Figure 5A**) revealed mainly pathways involved in metabolic processes (*e.g.*, amino acid metabolism, pyruvate metabolism) and pathways related to host immune responses (*e.g.*, signaling by interleukins). Pathways for DEPs higher in non-ICU patients were also related to metabolic processes (*e.g.*, aspartate metabolism, protein metabolic process) and host immune responses (e.g., antimicrobial response, cytokine-mediated signaling pathway) (**Figure 5B**). These findings showed that similar pathways were activated in all contrasts, which is not surprising because the main host response is directed towards a defense against the pathogen.

**Figure 6:**
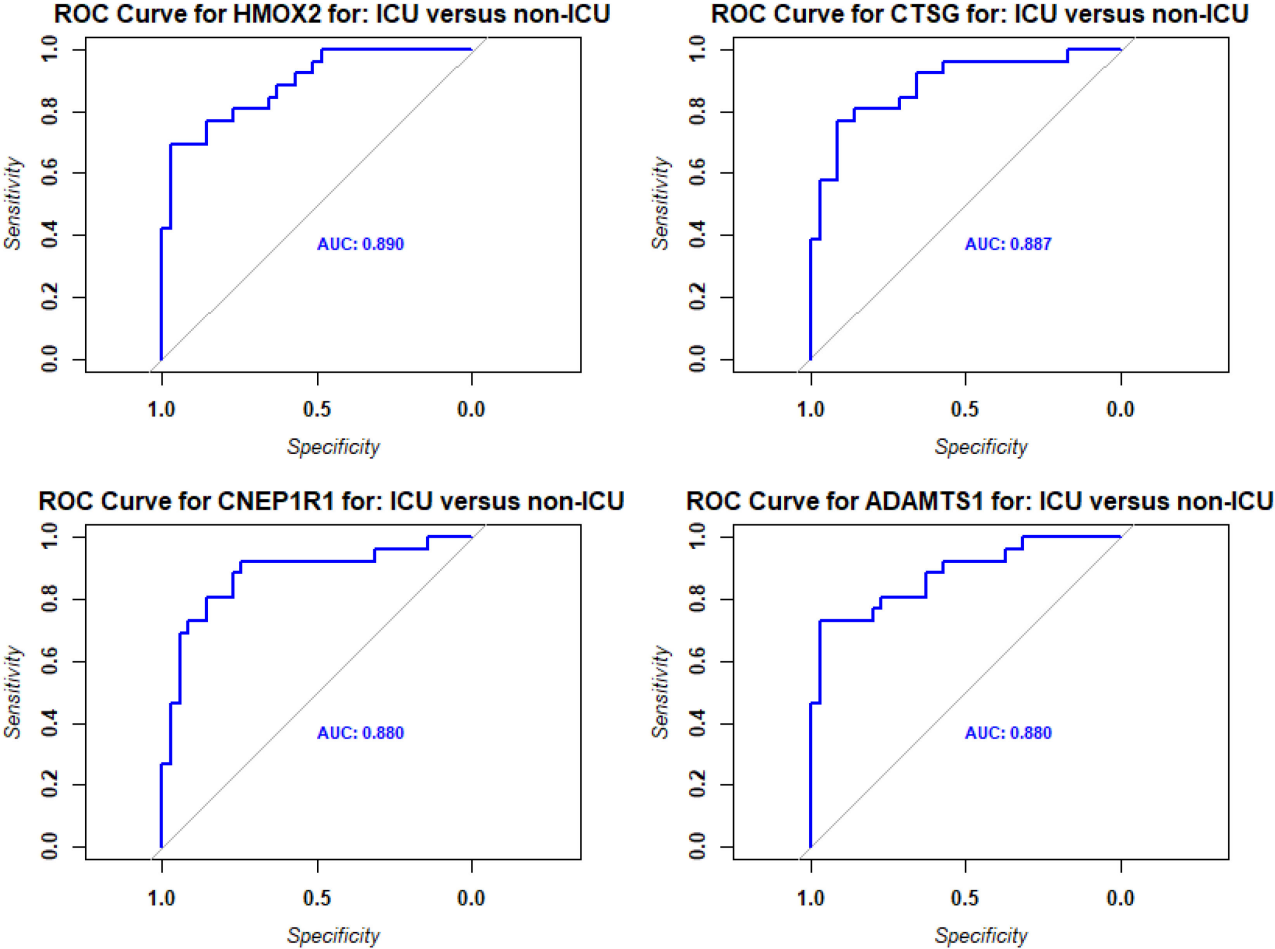
ROC analysis of DEPs from contrast of ICU versus non-ICU patients. Receiver operating characteristic (ROC) curves (blue) for the top four DEPs with the highest predictive values (AUC: area under the curve).

We then evaluated the predictive value of the DEPs by ROC analysis. No DEPs were identified with an AUC > 0.9. However, 75 DEPs exhibited a good AUC > 0.8; (**Supplementary Table S4**; **Figure 6** shows the top four DEPs with the highest predictive values).

### Age affects the responses in ICU patients

We then analyzed the effect of age in ICU patients. We contrasted old (> 65 years) to young (<= 65 years) patients in the ICU (8 old and 18 young cases) and identified 7 DEPs (1 up- and 6 down-regulated, **Supplementary Figure S3** Volcano; **Supplementary Table S2**). One of these DEPs (KNG1) overlapped with DEPs found for the contrast of ICU versus non-ICU cases.

### ICU patients have signatures of hyperinflammation and dysregulated cytokine regulation

**Figure 7:**
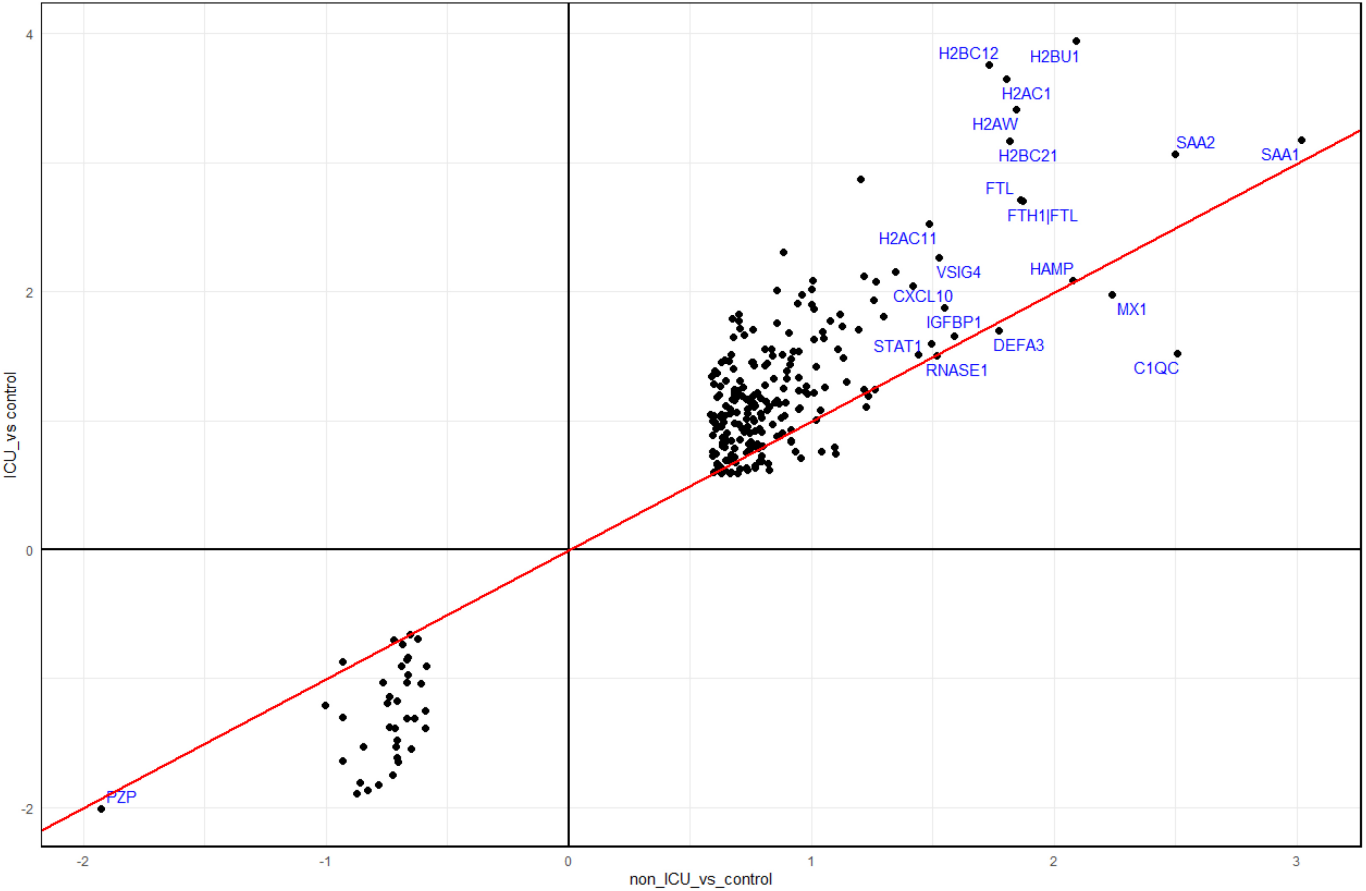
Comparison of DEPs from ICU and non-ICU patients versus controls. Scatter plot of DEPs from contrasts of ICU patients versus healthy controls and non-ICU patients versus healthy controls. Dots represent log_2_ differences of ICU versus controls (Y-axis) and non-ICU versus controls (x-axis). The top 20 proteins (by absolute expression levels in non-ICU) are labeled blue.

When comparing the responses in non-ICU and ICU patients to healthy controls, we identified many more DEPs in ICU patients (1213 in ICU versus 301 in non-ICU, **Supplementary Figure S4A**). The functional analyses of these DEPs revealed similar pathways for both contrasts (**Supplementary Figures S4B, C**). However, DEPs identified in both non-ICU and ICU patients (261 overlapping DEGs; **Supplementary Figure S4D**) showed a stronger response in ICU patients for both up- and downregulated DEPs (**Figure 7**). The functional analyses of these 261 overlapping DEPs also revealed immune response pathways (e.g., acute phase response, response to virus, inflammatory response, cytokine signaling, innate immune response, neutrophil degranulation, neutrophil extracellular trap formation) as a major response (**Supplementary Figure S4E**). In summary, these findings showed an increased immune response in ICU patients compared to non-ICU patients, which suggests a hyperinflammatory response in patients with severe disease. In addition, higher levels of histones were detected in the blood of ICU versus non-ICU patients (**Figure 4A**). This finding may indicate that ICU patients exhibit a stronger granulocyte response and release neutrophil extracellular traps (NETs) as a host defense in the lung composed of DNA, histones, and antimicrobial proteins. Components of these NETs may then appear in the peripheral blood.

**Figure 8:**
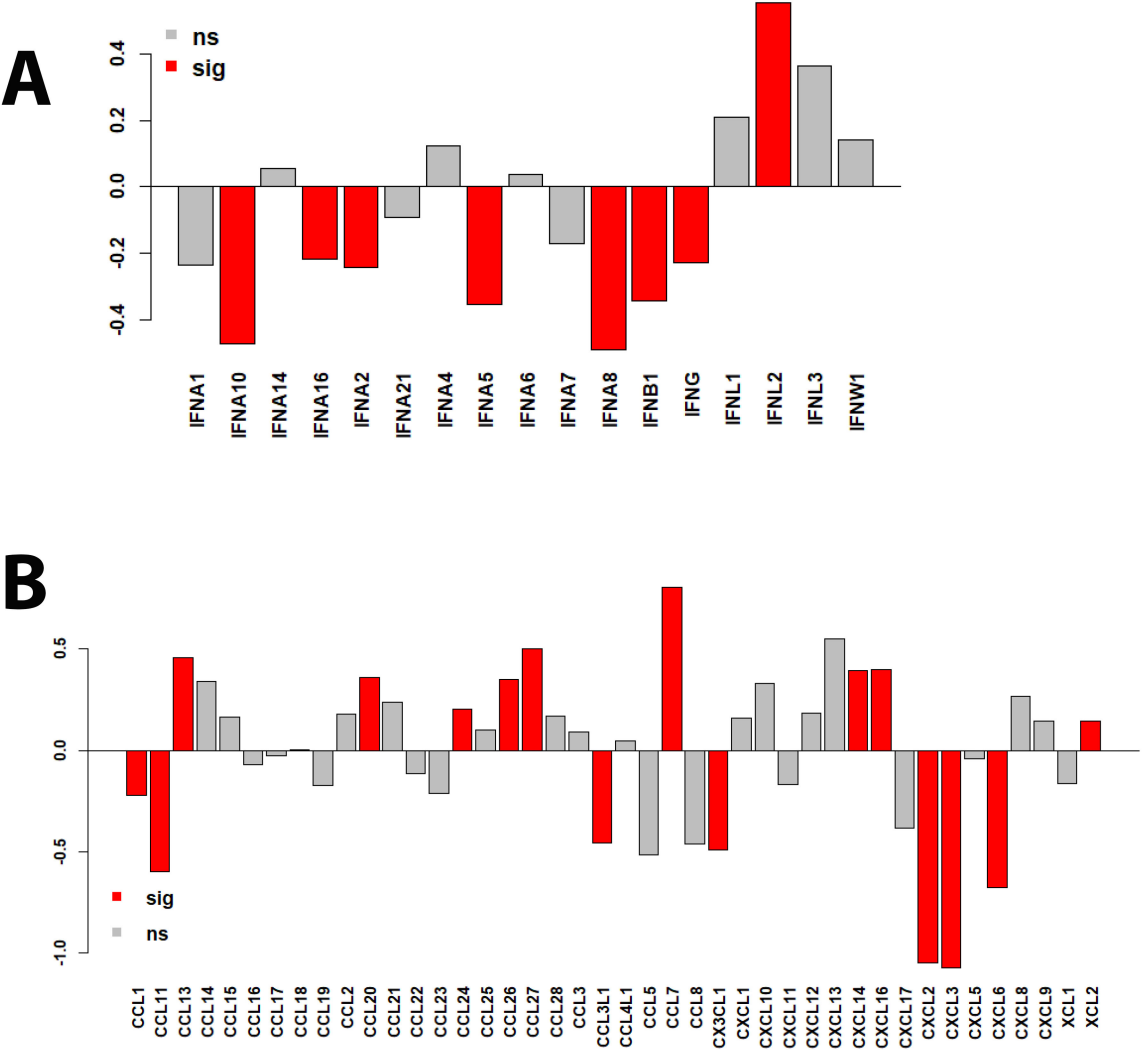
Bar plots of protein expression levels in ICU versus non-ICU patients. **(A)** Bar plots representing differences in log_2_ changes for ICU versus non-ICU patients for interferon proteins. **(B)** Bar plots representing differences in log_2_ changes for ICU versus non-ICU patients for chemokine and cytokine proteins. sig (red): DEPs that were significantly different (multiple testing BH-adjusted *P* values) in the contrast of ICU versus non-ICU patients. ns: not significant (grey).

We then investigated whether proteins from major innate immune response pathways were differently regulated between patients in the ICU and patients not in the ICU (non-ICU). For this, we used the interferon gene sets and chemokine/cytokine proteins listed in the human gene nomenclature (26). Most remarkably, most type I and II interferons were downregulated (7/13 reached statistical significance) in ICU versus non-ICU patients, whereas IFNL proteins were upregulated (**Figure 8A**). This was accompanied by nine cytokine/chemokine proteins being significantly upregulated (CCL13, CCL20, CCL24, CCL26, CCL7, CXCL14, CXCL16 and XCL2) and seven proteins significantly downregulated (CCL1, CCL11, CCL3L1, CX3CL1, CXCL2, CXCL3, and CXCL6) in ICU patients (**Figure 8B**), many of which are involved in macrophage and neutrophil recruitment.

### Many more DEPs identified compared to previous studies

In our study, many more not yet described set DEPs were detected compared to previous reports. Most previous studies only analyzed a few proteins using various antibodies as probes. Only two studies performed a comparable broad-coverage proteome analysis (1, 2). Both studies categorized patients in the ICU as having severe disease. The first study used the Bio-Plex Protein Array System with blood samples from 20 healthy controls, 26 patients with mild disease, and 15 patients with severe disease. They identified 16 DEPs that were significantly regulated in any group of infected, severe, and non-severe patients compared to healthy controls (see Table 2 in (2)). The second study used a multiplex Biorad 27-plex assay to compare 10 patients with severe disease versus 15 healthy controls and identified eight DEPs (1). The list of proteins from these studies is summarized in **Supplementary Table S7**. A set of 19 proteins was significantly regulated in mildly affected patients versus healthy controls in both studies. Of the 19 proteins, 13 were analyzed in our Somalogic panel. Two proteins, CXCL8 and CXCL10, were also significantly regulated for the contrast of non-ICU patients versus healthy controls in our study (**Supplementary Figure S5A**), with the direction (up or down) being the same. A set of 19 proteins was significantly regulated in severe patients versus healthy controls by both studies, of which 13 were also analyzed in our Somalogic panel for the ICU versus healthy controls contrast. Five of these 13 proteins, CXCL8, IFNG, IL13, IL6, and TNFSF10, were also significantly regulated in our study (**Supplementary Figure S5B**); the direction was the same for CXCL8, IL6, and TNFSF10. A set of 8 proteins was significantly regulated in severe versus mild patients by both studies, of which 6 were also analyzed in our SomaLogic panel for ICU versus healthy controls. Two proteins, IL12B and IL6, were also significantly regulated for this contrast in our study (**Supplementary Figure S5C**); the direction was the same for IL6.

### Correlation between blood proteome and cell transcriptome revealed new associations and crosstalk

**Figure 9:**
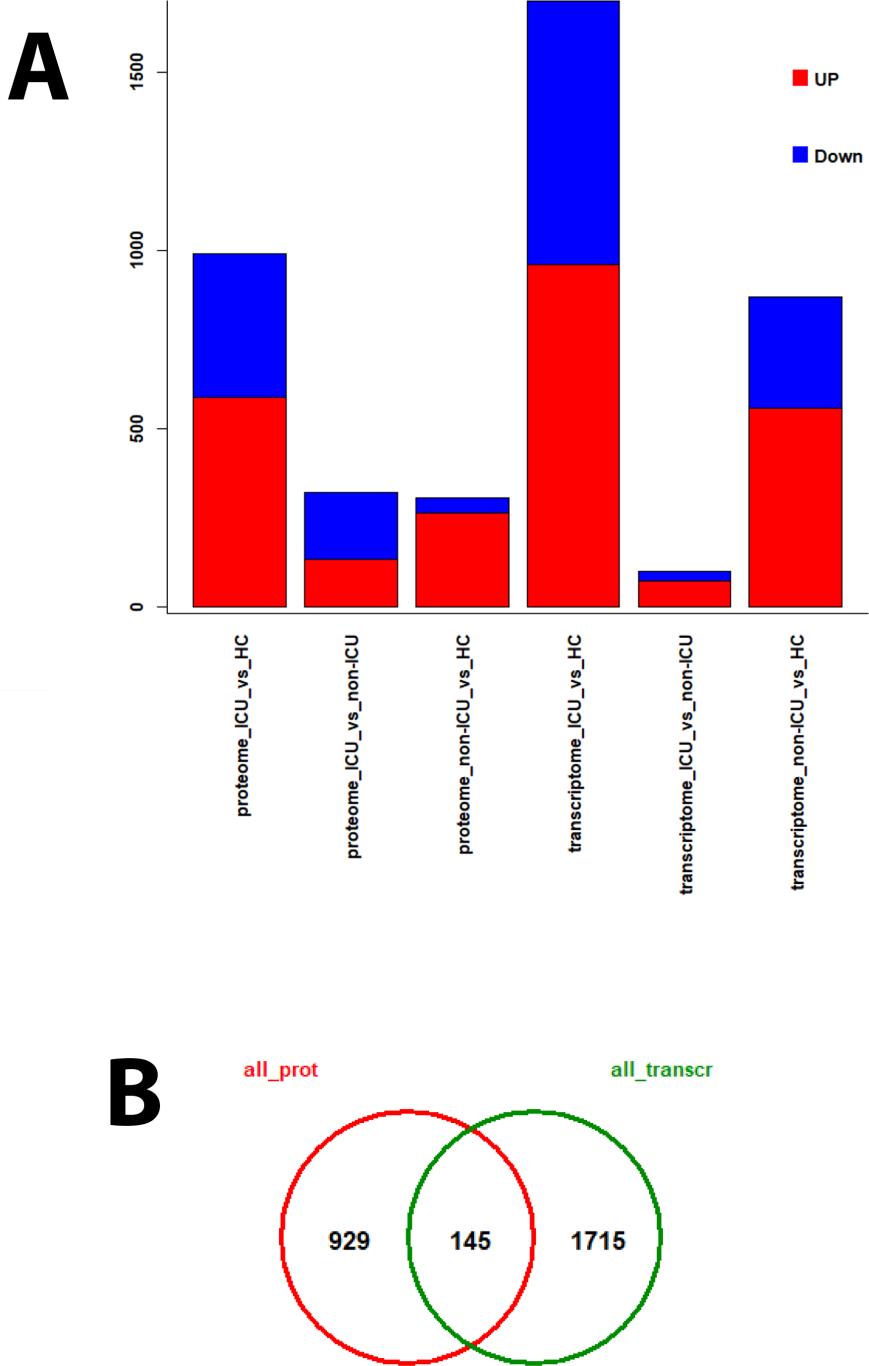
DEPs and DEGs for proteomes and transcriptomes from different contrasts. For patients from whom both transcriptome and proteome data were available, various contrasts for the identification of DEPs and DEGs were performed. **(A)** Bar plot representing the numbers of up- (red) and down-regulated (blue) DEPs and DEGs for the individual contrasts. **(B)** Venn diagram of overlapping DEPs (all_prot) and DEGs (all_transcr) using results from all contrasts.

We previously published transcriptomic analyses from the blood of the same patients (7) who were analyzed here for proteome changes (71 overlapping patients, details see M&M). Comparing the proteome to transcriptome expression levels identified fewer DEPs compared to DEGs for almost all contrasts (**Supplementary Table S8**, **Figure 9A**), which may be due to the methodology allowing us to detect many more transcripts than proteins. The VENN diagram comparing all DEPs with all DEGs (using non-ICU versus healthy controls + ICU versus healthy controls + ICU versus non-ICU contrasts) showed that 145 DEPs were found as DEGs in the transcriptome, whereas 929 were not (**Figure 9B**). This is an important finding because it demonstrates that the analysis of proteins in the serum detects unique biomarkers that are distinct from the blood cell transcriptome biomarkers.

Because proteomes and transcriptomes were studied in the same patient, we also looked for correlations between DEPs and related transcriptome gene expression levels. These results may allow for the generation or confirmation of causal hypotheses for proteins and genes, *e.g.*, the increased expression levels of a given interferon or cytokine in the serum may trigger a change in gene expression in blood cells. Using an adjusted *P* < 0.05 and a correlation coefficient of abs(> 0.6), we identified 597 DEPs showing significant correlation with at least one DEG (**Supplementary Table S9**). Using a higher correlation coefficient of abs(> 0.8) reduced this number to 27 DEPs with a significant correlation with at least one DEG (**Supplementary Table S9**).

**Figure 10:**
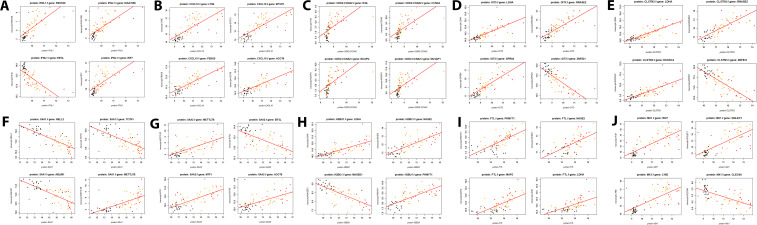
Scatter plots for proteins and their corresponding top four most strongly correlated DEGs. **(A)** IFNL1, **(B)** CXCL10, **(C)** CDK2 / CCNA2, **(D)** OIT3, **(E)** CLSTN3, **(F)** SAA1, **(G)** SAA2, **(H)** H2BU1, **(I)** FTL, and **(J)** MX1 proteins respectively, and their top four correlated genes. Dots represent transcriptome expression levels (Y-axis) and proteome expression levels (X-axis). Black: healthy controls, orange: non-ICU, and red: ICU cases.

The top 5 of these 27 DEPs with the highest number of correlated proteins were IFNL1 (15 correlated DEGs; **Supplementary Table S10**), CXCL10 (10 correlated DEGs; **Supplementary Table S10**), CDK2.CCNA2 (9 correlated DEGs; **Supplementary Table S10**), OIT3 (9 correlated DEGs; **Supplementary Table S10**), CLSTN3 (5 correlated DEGs; **Supplementary Table S10**). Scatter plots of the expression levels for these top DEPs and their most strongly correlated four top DEGs show tight correlation (**Figures 10A-E**). We also determined the correlations for the top five DEPs identified in the proteome contrast between infected versus healthy controls (considering only one histone: SAA1, SAA2, H2BU1, FTL, MX1) (**Figures 10F-J**, **Supplementary Table S10**). These results also demonstrate excellent correlations between the responses in the serum (proteome) and blood cells (transcriptome).

## Discussion

Understanding proteomic shifts during influenza virus infection, their correlation to cell transcriptomics, and the risk of ICU admission may provide new prognostic biomarkers and therapeutic targets. This study comprehensively examined the proteome in the blood of influenza-infected patients, some of whom had severe disease that required ICU admission.

Our analyses identified several DEPs in influenza-infected patients, when contrasting severity, most of which did not overlap with differentially expressed genes (DEGs) found in blood cell transcriptome studies (7, 9). Although some DEPs were correlated to DEGs, many more DEPs had not yet been described. Most of these changes were related to the host immune responses, metabolic reprogramming, chromatin remodeling, and iron metabolism.

Many DEPs exhibited very good (infected versus healthy controls) or good (ICU versus non-ICU) predictive values in the ROC analyses. Thus, our analyses identified many new potential biomarkers that distinguish responses in healthy versus infected and in ICU versus non-ICU patients, which likely reflect systemic changes and suggest several new signatures that may help predict severe disease and ICU admission. It should, however, be noted that our analysis only analyzed patients who were already in the ICU. Therefore, the markers that we identified will have to be tested in during progression of from moderate to severe disease to evaluate their clinical predictive value.

The hyperinflammatory response of ICU patients was accompanied by mixed cytokine signatures, possibly suggestive of a battle between inflammation and wound healing. There was an eosinophilic and fibrotic profile (increased CCL13 (MCP-4), CCL24 [Eotaxin-2], CCL26 [Eotaxin-3], CCL7 [MCP-3]; decreased CCL11 [Eotaxin-1]) and mixed T cell and macrophage recruitment (increased CCL20 [MIP-3α], CXCL14 [BRAK], CXCL16, XCL2; decreased CCL1, CCL3L1 [MIP-1α variant], and CX3CL1 [Fractalkine]), and suppressed neutrophil recruitment (decreased CXCL2 [MIP-2α], CXCL3, and CXCL6). These mixed Th2 and Th17 signatures may relate to SAA1 and SAA2, which display immunomodulatory functions in Th17 differentiation and macrophage polarization (27) in addition to having an important role in wound healing through FPR2-dependent epithelial migration (28). In addition, IL1RL1, which is involved in the IL-33-mediated signaling pathway (29, 30), was significantly different between ICU and non-ICU patients. Other studies have found similar proinflammatory signatures in patients with severe influenza, including elevated IL-8 (CXCL8), MCP-1 (CCL2), IP-10 (CXCL10), MIG (CXCL9), and MIP-1α (CCL3), and CD177 (10, 31). However, another study reported that H1N1 pandemic cases displayed suppressed adaptive cytokines (*e.g.*, IP-10 and MIG) (32), which may be due to differences in study design, cohort, and/or definition of case severity. Future studies will be needed to obtain more consistent data for the potential use of cytokines as biomarkers.

We also observed reduced levels of most type I and II interferon proteins in ICU patients compared to non-ICU patients, which aligns with an interferon response gene, MX1, being a top DEP. MX1 can negatively regulate viral genome replication (reviewed in (33)) and has a role in wound healing. The levels of IFNL proteins were increased in ICU patients, where IFNL2 was significantly higher, while IFNL1 and IFNL3 were higher but not significant. The latter observation could be indicative of higher viral loads as IFNL proteins represent a specific response to lung inflammation (34–36). Of note, IFNLs are thought to contribute to balancing tissue tolerance and conferring resistance to pathogen invasion (37, 38).

Numerous histones (H2BC12, H2BU1, H2AC1, and H2AW) were elevated in ICU patients compared to non-ICU patients. This may be a consequence of neutrophil extracellular traps (NETs), which are composed of DNA, histones, and antimicrobial proteins (39). An enhanced neutrophil response has been observed in transcriptome studies and shown to be correlated with severe outcomes (7–10, 40). Elevated levels of NET production have been observed in plasma from patients with severe influenza infections by measuring cell-free deoxyribonucleic acid (DNA) and myeloperoxidase (MPO)-DNA (41). SAA1 and SAA2, which are closely related to neutrophil responses (**Supplementary Figure S6**), were not significantly different between non-ICU and ICU patients, but they were most strongly expressed in infected patients compared to healthy controls. These proteins are also closely tied to apolipoproteins, which can influence the formation of NETs.

Proteins more highly expressed in non-ICU cases included mainly host immune responses. LRRC15 regulates protein localization to the plasma membrane. It is involved in the negative regulation of viral entry into host cells and receptor-mediated virion attachment to host cells (42, 43). TAPBPL is involved in the regulation of antigen processing and presentation of peptide antigen via MHC class I (44). CLEC4C is predicted to be involved in the innate immune response (45). IL36RN is involved in antifungal humoral response, negative regulation of cytokine production, and negative regulation of cytokine-mediated signaling pathways (46). The reduced expression of these proteins in ICU patients may indicate some compromised host responses that could lead to higher virus spread and then cause severe disease. These proteins may serve as biomarkers to diagnose or even to predict severe influenza infections. However, their usefulness and the best combinations will have to be further validated and confirmed in clinical settings.

We also analyzed the effect of age in ICU patients because an ANOVA analysis revealed an interaction of age and infection. CD177 was the only up-regulated DEP in patients older than 65 years. In our previous transcriptome analysis (10), we identified CD177 as the strongest up-regulated gene in blood cells of severe infections. The findings here confirm that the corresponding protein is also up-regulated in the serum of severe cases, especially in the elderly.

Many of the DEPs that we identified between infected and healthy controls have known functions related to immune responses, iron transport, and histones/chromatin modeling. The observed changes in metabolic pathways are similar to findings in pediatric studies, where an increase in glucose metabolism has been observed (47). Less is known about the influence of influenza infections on iron metabolism, where this study identified FTH1 and FTL as top DEPs, but it could be reflective of macrophage and/or IL-6 responses (48). However, a recent study in mice suggested that reducing iron availability may reduce viral replication, and influenza hemagglutinin has also been shown to trigger ferritinophagy, leading to iron-dependent cell death (49). It has been shown that influenza infection reduces the expression of genes for iron uptake proteins and decreases the expression of genes for iron storage proteins such as ferritin light (FTL) and heavy (FTH) chains (50).

Further, additional wound healing signatures were identified, including the DEPs MDK, which functions in the negative regulation of apoptotic processes, positive regulation of cell migration, and regulation of leukocyte cell-cell adhesion (51, 52), NTN1, which is involved in CDC42 protein signal transduction, plasma membrane-bounded cell projection organization, and positive regulation of axon extension (53–55), and FGFBP1, which represents a growth factor binding activity involved in positive regulation of blood vessel endothelial cell proliferation, sprouting angiogenesis, and positive regulation of cell migration (56). NTN1 is particularly interesting given its expression may indicate possible macrophage-related neuroinflammation (57).

A unique aspect of this study is that we analyzed the proteome and transcriptome of the same patients, which allowed us to perform correlations between proteins in the blood and gene expression in blood cells. The highest number of correlations was observed for IFNL1, an interferon that is strongly induced in the infected lung. Many studies have established that the activation of interferons, especially of type III, represents the major host response to viral infections, especially in the lung (34–37, 58–61). The genes highly correlated with the expression of IFNL1 protein included genes involved in interferon regulation (*IRF7, IFI6, IFI44*), anti-viral defense (*TRIM6, PLSCR1, LY6E, EIF3L*), host immune response (*IRF7, IFI6, IFI44, EIF3L, EIF2AK2*), and ubiquitination processes (*FBXO6, TRIM6, FBXO39*). Other genes were involved in various cellular processes not yet linked to the host response or anti-viral activities, like peptidase activity (*ST14*), mRNA binding activity (*TDRD7*), and translational elongation (GTPBP2) (46).

Another hallmark of a respiratory infection observed both in animal models and human studies is the expression of CXCL10 at both the transcriptome and the proteome level. CXCL10 stimulates monocyte, natural killer cell, and T cell migration, as well as modulation of adhesion molecule expression. It is thought to be a key regulator of the ‘cytokine storm’ induced after SARS-CoV-2 infection (62–66), and the same could be true for influenza. The genes highly correlated with the expression of CXCL10 protein (10 DEGs with coefficient > 0.8) in the host response to infections (*CCRL2, IFI6, IRF7, LY6E*) were genes involved in ubiquitination processes (*FBXO6*), and genes involved in various cellular processes not yet linked to the host response or anti-viral activities, like insertase activity (*TIMM10*), potassium:chloride symporter activity (*SLC12A8*), acetyl-CoA hydrolase activity (*ACOT9*). Of note, several of these DEGs overlapped with the IFNL1-correlated DEGs.

Our correlation analysis can also be used to identify DEPs that were correlated with specific DEGs or any protein with any gene. In our previous transcriptome analysis, we identified *MMP6* as the most strongly up-regulated DEG between ICU and non-ICU cases (7). Using a coefficient greater than 0.7 (no hits with a coefficient of 0.8), we found 15 DEPs correlated with the expression of this gene.

In conclusion, the DEPs identified in both non-ICU and ICU patients typically showed a stronger response in IUC patients for both up- and downregulated proteins, indicating that ICU patients exhibit a stronger inflammatory response than observed in non-ICU patients. Such hyperinflammatory immunopathogenic innate host immune responses have been described for severe courses following respiratory infections (67–69). The proteins identified here may serve as biomarkers, most likely if used in combinations, but their usefulness and the best combinations must be further validated and confirmed in clinical settings.

It should be noted that our study has several limitations. In human studies, there are always unknown confounding factors and differences in experimental outlines that may influence the results and conclusions, such as genetic and lifestyle heterogeneities in patient groups, collection methods and timing within clinics, and processing of samples. Therefore, results from subsequent studies should be compared to conclude whether our findings are consistent, and potential biomarkers must be validated. Nevertheless, we observed overlaps with other published studies, suggesting several prime candidates are worth evaluating further. Another limitation was that the effect of the collection site could not be analyzed because all healthy samples were collected at the Baptist Memorial Hospital. In addition, the influenza virus strain was not recorded. Many DEPs exhibited predictive values in ROC analyses. However, our analysis only analyzed patients who were already in the ICU. Therefore, the markers that we identified will have to be tested during the progression from moderate to severe disease. In summary, we identified many DEPs that may represent potential biomarkers. However, at this stage, we cannot suggest the best ones that would be suitable in clinical settings. Defining a short list will require more clinical testing and validation by additional methods (e.g., ELISA).

## Data Availability

The original contributions presented in the study are publicly available. This data can be found here: https://doi.org/10.6084/m9.figshare.27826857; https://doi.org/10.6084/m9.figshare.27827013; https://doi.org/10.6084/m9.figshare.27852273; https://doi.org/10.6084/m9.figshare.27852306; https://doi.org/10.6084/m9.figshare.29882222.v1; and https://doi.org/10.6084/m9.figshare.29882255.v1.

## References

[1] Bermejo-Martin JF Ortiz De Lejarazu R Pumarola T Rello J Almansa R Ramírez P . Th1 and Th17 hypercytokinemia as early host response signature in severe pandemic influenza. Crit Care. (2009) 13:R201. doi: 10.1186/cc8208, PMID: 20003352 PMC2811892

[2] Arankalle VA Lole KS Arya RP Tripathy AS Ramdasi AY Chadha MS . Role of host immune response and viral load in the differential outcome of pandemic H1N1 (2009) influenza virus infection in Indian patients. PloS One. (2010) 5. doi: 10.1371/journal.pone.0013099, PMID: 20957032 PMC2948498

[3] Teran LM Rüggeberg S Santiago J Fuentes-Arenas F Hernández JL Montes-Vizuet AR . Immune response to seasonal influenza A virus infection: a proteomic approach. Arch Med Res. (2012) 43:464–9. doi: 10.1016/j.arcmed.2012.08.008, PMID: 22960859

[4] Marion T Elbahesh H Thomas PG Devincenzo JP Webby R Schughart K . Respiratory mucosal proteome quantification in human influenza infections. PloS One. (2016) 11:e0153674. doi: 10.1371/journal.pone.0153674, PMID: 27088501 PMC4835085

[5] Sande CJ Mutunga M Muteti J Berkley JA Nokes DJ Njunge J . Untargeted analysis of the airway proteomes of children with respiratory infections using mass spectrometry based proteomics. Sci Rep. (2018) 8:13814. doi: 10.1038/s41598-018-32072-3, PMID: 30217988 PMC6138648

[6] Lydon E Osborne CM Wagner BD Ambroggio L Harris JK Reeder R . Proteomic profiling of the local and systemic immune response to pediatric respiratory viral infections. mSystems. (2025) 10:e0133524. doi: 10.1128/msystems.01335-24, PMID: 39611811 PMC11748518

[7] Schughart K Smith AM Tsalik EL Threlkeld SC Sellers S Fischer WA 2nd Schreiber J . Host response to influenza infections in human blood: association of influenza severity with host genetics and transcriptomic response. Front Immunol. (2024) 15:1385362. doi: 10.3389/fimmu.2024.1385362, PMID: 39192977 PMC11347429

[8] Bermejo-Martin JF Martin-Loeches I Rello J Anton A Almansa R Xu L . Host adaptive immunity deficiency in severe pandemic influenza. Crit Care. (2010) 14:R167. doi: 10.1186/cc9259, PMID: 20840779 PMC3219262

[9] Dunning J Blankley S Hoang LT Cox M Graham CM James PL . Progression of whole-blood transcriptional signatures from interferon-induced to neutrophil-associated patterns in severe influenza. Nat Immunol. (2018) 19:625–35. doi: 10.1038/s41590-018-0111-5, PMID: 29777224 PMC5985949

[10] Tang BM Shojaei M Teoh S Meyers A Ho J Ball TB . Neutrophils-related host factors associated with severe disease and fatality in patients with influenza infection. Nat Commun. (2019) 10:3422. doi: 10.1038/s41467-019-11249-y, PMID: 31366921 PMC6668409

[11] Gold L Ayers D Bertino J Bock C Bock A Brody EN . (2010). Aptamer-basedmultiplexed proteomic technology for biomarker discovery. PLoS One 5, e15004. doi: 10.1371/journal.pone.0015004, PMID: 21165148 PMC3000457

[12] Han Z Xiao Z Kalantar-Zadeh K Moradi H Shafi T Waikar SS . Validation of a novel modified aptamer-based array proteomic platform in patients with end-stage renal disease. Diagn (Basel). (2018) 8. doi: 10.3390/diagnostics8040071, PMID: 30297602 PMC6316431

[13] Tin A Yu B Ma J Masushita K Daya N Hoogeveen RC . Reproducibility and variability of protein analytes measured using a multiplexed modified aptamer assay. J Appl Lab Med. (2019) 4:30–9. doi: 10.1373/jalm.2018.027086, PMID: 31639705 PMC6814271

[14] Yang J Brody EN Murthy AC Mehler RE Weiss SJ Delisle RK . Impact of kidney function on the blood proteome and on protein cardiovascular risk biomarkers in patients with stable coronary heart disease. J Am Heart Assoc. (2020) 9:e016463. doi: 10.1161/JAHA.120.016463, PMID: 32696702 PMC7792282

[15] Smyth GK . Linear models and empirical bayes methods for assessing differential expression in microarray experiments. Stat Appl Genet Mol Biol. (2004) 3:Article3. doi: 10.2202/1544-6115.1027, PMID: 16646809

[16] Ritchie ME Phipson B Wu D Hu Y Law CW Shi W . limma powers differential expression analyses for RNA-sequencing and microarray studies. Nucleic Acids Res. (2015) 43:e47. doi: 10.1093/nar/gkv007, PMID: 25605792 PMC4402510

[17] R_Core_Team . R: A language and environment for statistical computing. R foundation for statistical computing. Austria: Vienna (2013). Available online at: http://www.R-project.org/ (Accessed November 07, 2025).

[18] Rstudio . RStudio website . Available online at: https://www.rstudio.com/ (Accessed November 07, 2025).

[19] Blighe K Rana S Lewis M . EnhancedVolcano: Publication-ready volcano plots with enhanced colouring and labeling . Available online at: https://github.com/kevinblighe/EnhancedVolcano (Accessed November 07, 2025).

[20] Chen EY Tan CM Kou Y Duan Q Wang Z Meirelles GV . Enrichr: interactive and collaborative HTML5 gene list enrichment analysis tool. BMC Bioinf. (2013) 14:128. doi: 10.1186/1471-2105-14-128, PMID: 23586463 PMC3637064

[21] Kuleshov MV Jones MR Rouillard AD Fernandez NF Duan Q Wang Z . Enrichr: a comprehensive gene set enrichment analysis web server 2016 update. Nucleic Acids Res. (2016) 44:W90–97. doi: 10.1093/nar/gkw377, PMID: 27141961 PMC4987924

[22] Xie Z Bailey A Kuleshov MV Clarke DJB Evangelista JE Jenkins SL . Gene set knowledge discovery with enrichr. Curr Protoc. (2021) 1:e90. doi: 10.1002/cpz1.90, PMID: 33780170 PMC8152575

[23] Eklund A . beeswarm: the beeSwarm plot, an alternative to stripchart (2016). Available online at: https://CRAN.R-project.org/package=beeswarm (Accessed November 07, 2025).

[24] Benjamini Y Hochberg Y . Controlling the false discovery rate: A practical and powerful approach to multiple testing. J R Stat Soc. (1995) 57:289–300. doi: 10.1111/j.2517-6161.1995.tb02031.x

[25] Hutchins AP Poulain S Miranda-Saavedra D . Genome-wide analysis of STAT3 binding *in vivo* predicts effectors of the anti-inflammatory response in macrophages. Blood. (2012) 119:e110–119. doi: 10.1182/blood-2011-09-381483, PMID: 22323479

[26] Hgnc . Human gene nomenclature . Available online at: hMps://www.genenames.org/ (Accessed November 07, 2025).

[27] Chang Y Liu Y Zou Y Ye RD . Recent advances in studies of serum amyloid A: implications in inflammation, immunity and tumor metastasis. Int J Mol Sci. (2025) 26. doi: 10.3390/ijms26030987, PMID: 39940756 PMC11817213

[28] Hinrichs BH Matthews JD Siuda D O’leary MN Wolfarth AA Saeedi BJ . Serum amyloid A1 is an epithelial prorestitutive factor. Am J Pathol. (2018) 188:937–49. doi: 10.1016/j.ajpath.2017.12.013, PMID: 29366677 PMC5866105

[29] Ho JE Chen WY Chen MH Larson MG Mccabe EL Cheng S . Common genetic variation at the IL1RL1 locus regulates IL-33/ST2 signaling. J Clin Invest. (2013) 123:4208–18. doi: 10.1172/JCI67119, PMID: 23999434 PMC3784527

[30] Pinto SM Subbannayya Y Rex D Raju R Chatterjee O Advani J . A network map of IL-33 signaling pathway. J Cell Commun Signal. (2018) 12:615–24. doi: 10.1007/s12079-018-0464-4, PMID: 29705949 PMC6039344

[31] Gu Y Hsu AC Pang Z Pan H Zuo X Wang G . Role of the innate cytokine storm induced by the influenza A virus. Viral Immunol. (2019) 32:244–51. doi: 10.1089/vim.2019.0032, PMID: 31188076

[32] Garcinuño S Lalueza A Gil-Etayo FJ Díaz-Simón R Lizasoain I Moraga A . Immune dysregulation is an important factor in the underlying complications in Influenza infection. ApoH, IL-8 and IL-15 as markers of prognosis. Front Immunol. (2024) 15:1443096. doi: 10.3389/fimmu.2024.1443096, PMID: 39176097 PMC11339618

[33] Haller O Staeheli P Schwemmle M Kochs G . Mx GTPases: dynamin-like antiviral machines of innate immunity. Trends Microbiol. (2015) 23:154–63. doi: 10.1016/j.tim.2014.12.003, PMID: 25572883

[34] Jewell NA Cline T Mertz SE Smirnov SV Flaño E Schindler C . Lambda interferon is the predominant interferon induced by influenza A virus infection in *vivo*. J Virol. (2010) 84:11515–22. doi: 10.1128/JVI.01703-09, PMID: 20739515 PMC2953143

[35] Galani IE Triantafyllia V Eleminiadou EE Koltsida O Stavropoulos A Manioudaki M . Interferon-λ Mediates Non-redundant Front-Line Antiviral Protection against Influenza Virus Infection without Compromising Host Fitness. Immunity. (2017) 46:875–890.e876. doi: 10.1016/j.immuni.2017.04.025, PMID: 28514692

[36] Wells AI Coyne CB . Type III interferons in antiviral defenses at barrier surfaces. Trends Immunol. (2018) 39:848–58. doi: 10.1016/j.it.2018.08.008, PMID: 30219309 PMC6179363

[37] Broggi A Granucci F Zanoni I . Type III interferons: Balancing tissue tolerance and resistance to pathogen invasion. J Exp Med. (2020) 217. doi: 10.1084/jem.20190295, PMID: 31821443 PMC7037241

[38] Antos D Alcorn JF . IFNλ: balancing the light and dark side in pulmonary infection. mBio. (2023) 14:e0285022. doi: 10.1128/mbio.02850-22, PMID: 37278532 PMC10470512

[39] Wang Y Du C Zhang Y Zhu L . Composition and function of neutrophil extracellular traps. Biomolecules. (2024) 14. doi: 10.3390/biom14040416, PMID: 38672433 PMC11048602

[40] Kaplan MJ Radic M . Neutrophil extracellular traps: double-edged swords of innate immunity. J Immunol. (2012) 189:2689–95. doi: 10.4049/jimmunol.1201719, PMID: 22956760 PMC3439169

[41] Zhu L Liu L Zhang Y Pu L Liu J Li X . High level of neutrophil extracellular traps correlates with poor prognosis of severe influenza A infection. J Infect Dis. (2018) 217. 428–437> doi: 10.1093/infdis/jix475, PMID: 29325098

[42] Song J Chow RD Peña-Hernández MA Zhang L Loeb SA So EY . LRRC15 inhibits SARS-CoV-2 cellular entry in trans. PloS Biol. (2022) 20:e3001805. doi: 10.1371/journal.pbio.3001805, PMID: 36228039 PMC9595563

[43] Shilts J Crozier TWM Teixeira-Silva A Gabaev I Gerber PP Greenwood EJD . LRRC15 mediates an accessory interaction with the SARS-CoV-2 spike protein. PloS Biol. (2023) 21:e3001959. doi: 10.1371/journal.pbio.3001959, PMID: 36735681 PMC9897555

[44] Hermann C Trowsdale J Boyle LH . TAPBPR: a new player in the MHC class I presentation pathway. Tissue Antigens. (2015) 85:155–66. doi: 10.1111/tan.12538, PMID: 25720504

[45] Riboldi E Daniele R Parola C Inforzato A Arnold PL Bosisio D . Human C-type lectin domain family 4, member C (CLEC4C/BDCA-2/CD303) is a receptor for asialo-galactosyl-oligosaccharides. J Biol Chem. (2011) 286:35329–33. doi: 10.1074/jbc.C111.290494, PMID: 21880719 PMC3195614

[46] Alliance_of_Genome_Resources . Alliance of genome resources. Available online at: https://www.alliancegenome.org/ (Accessed November 07, 2025).

[47] Smallwood HS Duan S Morfouace M Rezinciuc S Shulkin BL Shelat A . Targeting metabolic reprogramming by influenza infection for therapeutic intervention. Cell Rep. (2017) 19:1640–53. doi: 10.1016/j.celrep.2017.04.039, PMID: 28538182 PMC5599215

[48] Fan Y Zhang J Cai L Wang S Liu C Zhang Y . The effect of anti-inflammatory properties of ferritin light chain on lipopolysaccharide-induced inflammatory response in murine macrophages. Biochim Biophys Acta. (2014) 1843:2775–83. doi: 10.1016/j.bbamcr.2014.06.015, PMID: 24983770

[49] Ouyang A Chen T Feng Y Zou J Tu S Jiang M . The hemagglutinin of influenza A virus induces ferroptosis to facilitate viral replication. Adv Sci (Weinh). (2024) 11:e2404365. doi: 10.1002/advs.202404365, PMID: 39159143 PMC11497066

[50] Mayall J Pillar A Daly K . Crucial role of iron metabolism in determining outcomes of influenza A virus infection and disease. Eur Respir J. (2022) 60:877. doi: 10.1183/13993003.congress-2022.877

[51] Muramatsu T Kadomatsu K . Midkine: an emerging target of drug development for treatment of multiple diseases. Br J Pharmacol. (2014) 171:811–3. doi: 10.1111/bph.12571, PMID: 24460672 PMC3925019

[52] Weckbach LT Gola A Winkelmann M Jakob SM Groesser L Borgolte J . The cytokine midkine supports neutrophil trafficking during acute inflammation by promoting adhesion via β2 integrins (CD11/CD18). Blood. (2014) 123:1887–96. doi: 10.1182/blood-2013-06-510875, PMID: 24458438

[53] Serafini T Kennedy TE Galko MJ Mirzayan C Jessell TM Tessier-Lavigne M . The netrins define a family of axon outgrowth-promoting proteins homologous to C. elegans UNC-6. Cell. (1994) 78:409–24. doi: 10.1016/0092-8674(94)90420-0, PMID: 8062384

[54] Shekarabi M Kennedy TE . The netrin-1 receptor DCC promotes filopodia formation and cell spreading by activating Cdc42 and Rac1. Mol Cell Neurosci. (2002) 19:1–17. doi: 10.1006/mcne.2001.1075, PMID: 11817894

[55] Shekarabi M Moore SW Tritsch NX Morris SJ Bouchard JF Kennedy TE . Deleted in colorectal cancer binding netrin-1 mediates cell substrate adhesion and recruits Cdc42, Rac1, Pak1, and N-WASP into an intracellular signaling complex that promotes growth cone expansion. J Neurosci. (2005) 25:3132–41. doi: 10.1523/JNEUROSCI.1920-04.2005, PMID: 15788770 PMC6725078

[56] Chen X Miao M Zhou M Chen J Li D Zhang L . Poly-L-arginine promotes asthma angiogenesis through induction of FGFBP1 in airway epithelial cells via activation of the mTORC1-STAT3 pathway. Cell Death Dis. (2021) 12:761. doi: 10.1038/s41419-021-04055-2, PMID: 34341336 PMC8329163

[57] Gu L Zhou Y Wang G Deng H Song X He X . Spatial learning and memory impaired after infection of non-neurotropic influenza virus in BALB/c male mice. Biochem Biophys Res Commun. (2021) 540:29–36. doi: 10.1016/j.bbrc.2020.12.092, PMID: 33429197

[58] Mordstein M Kochs G Dumoutier L Renauld JC Paludan SR Klucher K . Interferon-lambda contributes to innate immunity of mice against influenza A virus but not against hepatotropic viruses. PloS Pathog. (2008) 4:e1000151. doi: 10.1371/journal.ppat.1000151, PMID: 18787692 PMC2522277

[59] Crotta S Davidson S Mahlakoiv T Desmet CJ Buckwalter MR Albert ML . Type I and type III interferons drive redundant amplification loops to induce a transcriptional signature in influenza-infected airway epithelia. PloS Pathog. (2013) 9:e1003773. doi: 10.1371/journal.ppat.1003773, PMID: 24278020 PMC3836735

[60] Hemann EA Gale M Jr. Savan R . Interferon lambda genetics and biology in regulation of viral control. Front Immunol. (2017) 8:1707. doi: 10.3389/fimmu.2017.01707, PMID: 29270173 PMC5723907

[61] Lozhkov AA Klotchenko SA Ramsay ES Moshkoff HD Moshkoff DA Vasin AV . The key roles of interferon lambda in human molecular defense against respiratory viral infections. Pathogens. (2020) 9. doi: 10.3390/pathogens9120989, PMID: 33255985 PMC7760417

[62] Christensen JE De Lemos C Moos T Christensen JP Thomsen AR . CXCL10 is the key ligand for CXCR3 on CD8+ effector T cells involved in immune surveillance of the lymphocytic choriomeningitis virus-infected central nervous system. J Immunol. (2006) 176:4235–43. doi: 10.4049/jimmunol.176.7.4235, PMID: 16547260

[63] Petrovic-Djergovic D Popovic M Chittiprol S Cortado H Ransom RF Partida-Sánchez S . CXCL10 induces the recruitment of monocyte-derived macrophages into kidney, which aggravate puromycin aminonucleoside nephrosis. Clin Exp Immunol. (2015) 180:305–15. doi: 10.1111/cei.12579, PMID: 25561167 PMC4408165

[64] Zhao Q Kim T Pang J Sun W Yang X Wang J . A novel function of CXCL10 in mediating monocyte production of proinflammatory cytokines. J Leukoc Biol. (2017) 102:1271–80. doi: 10.1189/jlb.5A0717-302, PMID: 28899907

[65] Callahan V Hawks S Crawford MA Lehman CW Morrison HA Ivester HM . The pro-inflammatory chemokines CXCL9, CXCL10 and CXCL11 are upregulated following SARS-coV-2 infection in an AKT-dependent manner. Viruses. (2021) 13. doi: 10.3390/v13061062, PMID: 34205098 PMC8226769

[66] Gudowska-Sawczuk M Mroczko B . What is currently known about the role of CXCL10 in SARS-coV-2 infection? Int J Mol Sci. (2022) 23. doi: 10.3390/ijms23073673, PMID: 35409036 PMC8998241

[67] Liu Q Zhou YH Yang ZQ . The cytokine storm of severe influenza and development of immunomodulatory therapy. Cell Mol Immunol. (2016) 13:3–10. doi: 10.1038/cmi.2015.74, PMID: 26189369 PMC4711683

[68] Silva MJA Ribeiro LR Gouveia MIM Marcelino BDR Santos CSD Lima KVB . Hyperinflammatory response in COVID-19: A systematic review. Viruses. (2023) 15. doi: 10.3390/v15020553, PMID: 36851766 PMC9962879

[69] Yunis J Short KR Yu D . Severe respiratory viral infections: T-cell functions diverging from immunity to inflammation. Trends Microbiol. (2023) 31:644–56. doi: 10.1016/j.tim.2022.12.008, PMID: 36635162 PMC9829516

