# Supplementary material for: Analysis of blood proteome in influenza-infected patients reveals new insights into the host response signatures distinguishing mild severe infections
Source: Front Immunol. 2025 Nov 19;16:1693728. doi: 10.3389/fimmu.2025.1693728 (PMC12672548; doi:10.3389/fimmu.2025.1693728)

Title page

Analysis of blood proteome in influenza-infected patients reveals new insights into the host response and novel signatures distinguishing mild and severe infections

Klaus Schughart[1,2,*], Stephen C. Threlkeld[3], Subhashini A. Sellers[4], William A. Fischer II[4], Jens Schreiber[5], Eva Lücke[5], Mark Heise[6,7], Amber M Smith[2,8]
[1]Institute of Virology Münster, University of Münster, Münster, Germany
[2]Department of Microbiology, Immunology and Biochemistry, University of Tennessee Health Science Center, Memphis, TN, United States
[3]Baptist Memorial Hospital, Memphis, TN, United States
[4]Division of Pulmonary Diseases and Critical Care Medicine, Department of Medicine, University of North Carolina at Chapel Hill, Chapel Hill, NC, United States
[5]Clinic of Pneumology, Otto-von-Guerike University, Magdeburg, Germany
[6]Department of Genetics, University of North Carolina at Chapel Hill, Chapel Hill, NC, United States
[7]Department of Microbiology and Immunology, University of North Carolina at Chapel Hill, Chapel Hill, NC, United States
[8]Department of Pediatrics, University of Tennessee Health Science Center, Memphis, TN, United States

***Correspondence:**
Name: Klaus Schughart
Email: labschughart@online.de
Address: Institute of Virology Münster, University of Münster, Von-Esmarch-Str. 56, 48149 Münster, Germany

Keywords
Influenza, proteome, DEPs, correlations, transcriptome

Figure S1A: String network analysis of 200 most strongly regulated DEPs from contrast of infected patients versus healthy controls.
String network analysis using STRING interactive website (https://string-db.org/cgi/input?sessionId=bZa9VJumLnb8&input_page_show_search=on) using basic settings: full STRING network and evidence = true. Colored nodes represent query proteins, filled nodes: a 3D structure is known or predicted. Bluegreen edges: protein-protein associations from curated databases, magenta edges: protein-protein associations from experimentally determined results, green edges: predicted protein-protein associations from gene neighborhoods, red edges: predicted protein-protein associations from gene fusions, blue edges: predicted protein-protein associations from gene co-occurrences, light green edges: predicted protein-protein associations from text mining, black edges: predicted protein-protein associations from gene co-expression, light blue edges: predicted protein-protein associations from protein homology. Colors of nodes refer to the same characteristics as described above for edges.

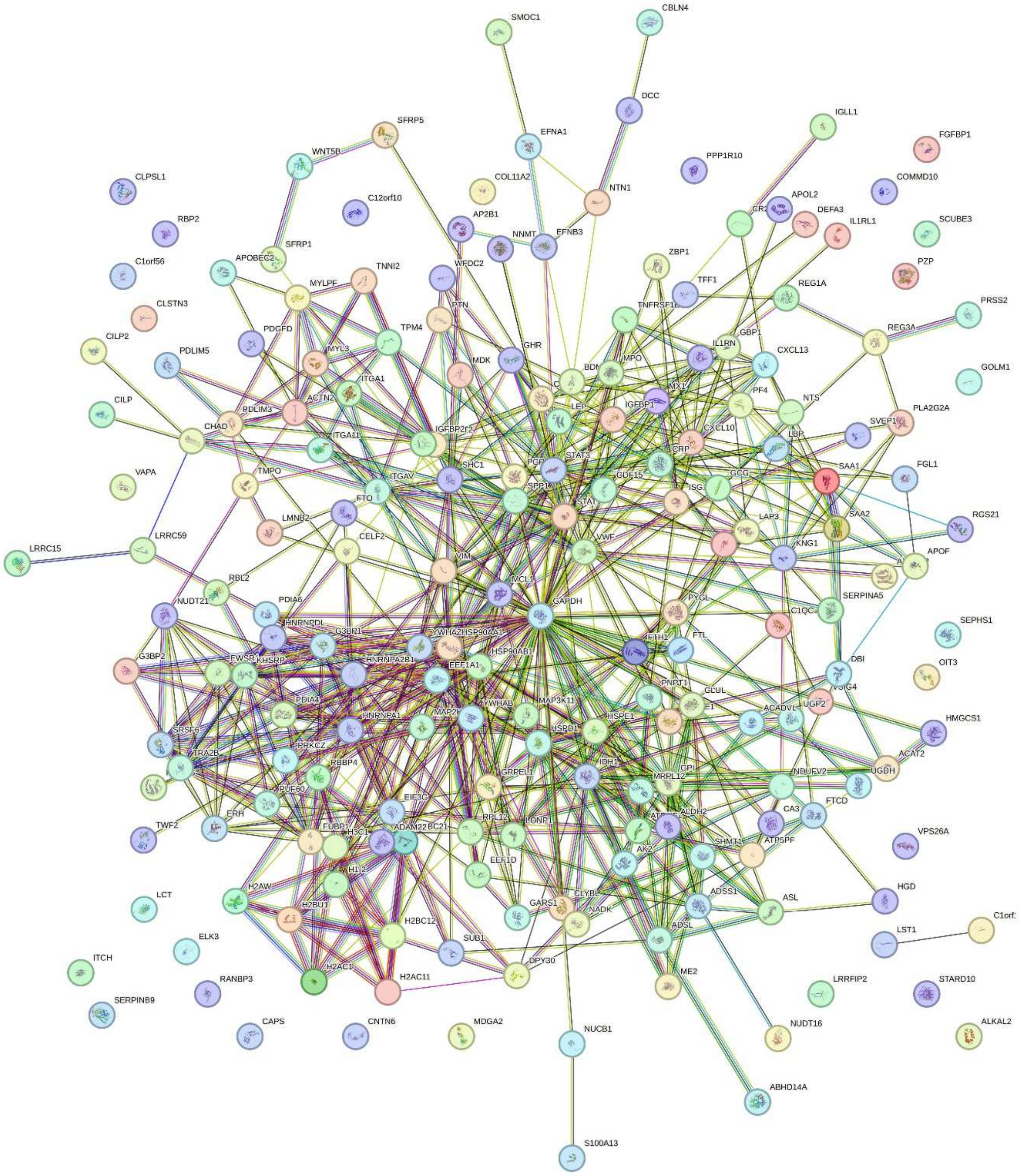

SMOC1
CBLN4
DCC
SFRP5
IGLL1
EFNA1
FGFBP1
PPP1R10
WNT5B
CLPSL1
COL11A2
COMMD10
NTN1
C12orf10
APOL2
RBP2
AP2B1
DEFA3
SCUBE3
IL1RL1
NNMT
EFNB3
SFRP1
C1orf56
APOBEC2
ZBP1
WFDC2
PZP
TNNI2
TFF1
MYLPF
REG1A
CLSTN3
PTN
PRSS2
TNFRSF1B
CILP2
PDGFD
TPM4
REG3A
MYL3
GBP1
PDLIM5
MDK
GHR
IL1RN
CXCL13
GOLM1
ITGA1
MPO
MX1
PF4
ACTN2
NTS
PLA2G2A
CILP
IGFBP1
LEP
PDLIM3
CHAD
CXCL10
SVEP1
ITGA11
IGFBP2
CRP
STAT3
LBP
SHC1
VAPA
TMPO
GDF15
GCG
SAA1
FGL1
ITGAV
SPP1
ISG15
FTO
LMNB2
LAP3
SAA2
RGS21
CELF2
VWF
KNG1
LRRC59
APOF
LRRC15
VIM
RBL2
MCL1
PYGL
SERPINA5
PDIA6
NUDT21
GAPDH
C1QC
HNRNPDL
G3BP1
YWHAZ
HSP90AA1
FTH1
FTL
SEPHS1
HSP90AB1
G3BP2
FUS
KHSRP
HNRNPA2B1
EEF1A1
DBI
OIT3
PNPT1
GLUL
VSIG4
PDIA4
MAP3K11
UGP2
MAP2
YWHAB
ACADVL
HNRNPA1
HSPE1
HMGCS1
SRSF6
LSR
PRKCZ
TRA2B
RBBP4
IDH1
ACAT2
GPI
UGDH
NDUFV2
MRPL12
PUF60
GRPEL1
FTCD
EIF3C
ALDH2
CA3
ERH
TWF2
LONP1
RPL12
FUBP1
ADAM22
H3C1
ATP5PF
SHMT1
VPS26A
AK2
EEF1D
H1-2
CLYBL
H2AW
HGD
GARS1
NADK
ADSS1
ASL
LCT
H2BU1
H2BC12
C1orf
LST1
ADSL
ELK3
SUB1
ITCH
H2AC11
DPY30
H2AC1
RANBP3
ME2
LRRFIP2
STARD10
SERPINB9
NUDT16
CAPS
CNTN6
MDGA2
ALKAL2
NUCB1
ABHD14A
S100A13

Figure S1B: String network analysis of proteins interacting with SAA1 & SAA2.
String network analysis using STRING interactive website (https://string-db.org/cgi/input?sessionId=bZa9VJumLnb8&input_page_show_search=on) using basic settings: full STRING network and evidence = true. Colored nodes represent query proteins, filled nodes: a 3D structure is known or predicted. Bluegreen edges: protein-protein associations from curated databases, magenta edges: protein-protein associations from experimentally determined results, green edges: predicted protein-protein associations from gene neighborhoods, red edges: predicted protein-protein associations from gene fusions, blue edges: predicted protein-protein associations from gene co-occurrences, light green edges: predicted protein-protein associations from text mining, black edges: predicted protein-protein associations from gene co-expression, light blue edges: predicted protein-protein associations from protein homology. Colors of nodes refer to the same characteristics as described above for edges.

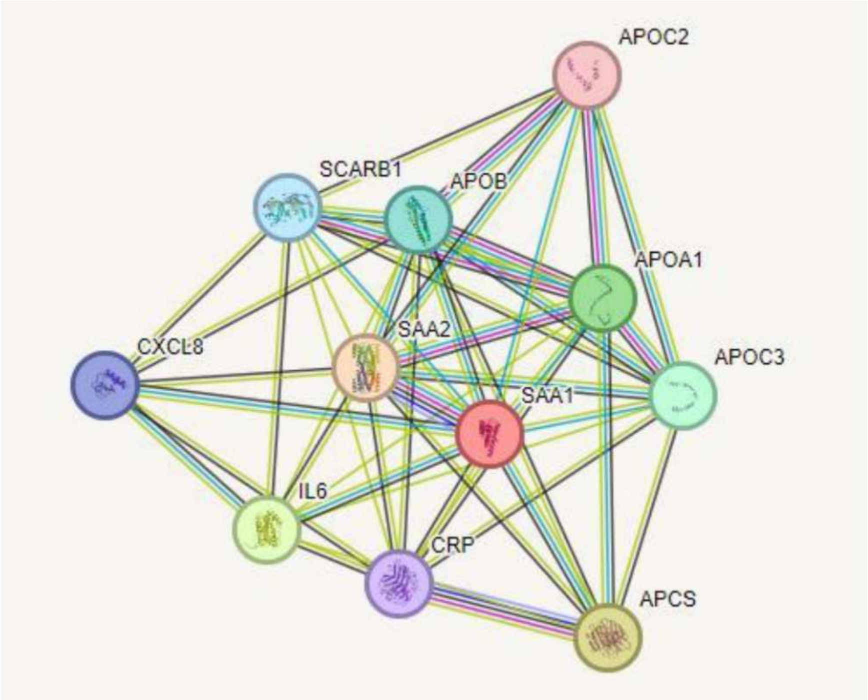

Figure S1C: String network analysis of proteins interacting with H2BU1.
String network analysis using STRING interactive website (https://string-db.org/cgi/input?sessionId=bZa9VJumLnb8&input_page_show_search=on) using basic settings: full STRING network and evidence = true. Colored nodes represent query proteins, filled nodes: a 3D structure is known or predicted. Bluegreen edges: protein-protein associations from curated databases, magenta edges: protein-protein associations from experimentally determined results, green edges: predicted protein-protein associations from gene neighborhoods, red edges: predicted protein-protein associations from gene fusions, blue edges: predicted protein-protein associations from gene co-occurrences, light green edges: predicted protein-protein associations from text mining, black edges: predicted protein-protein associations from gene co-expression, light blue edges: predicted protein-protein associations from protein homology. Colors of nodes refer to the same characteristics as described above for edges.

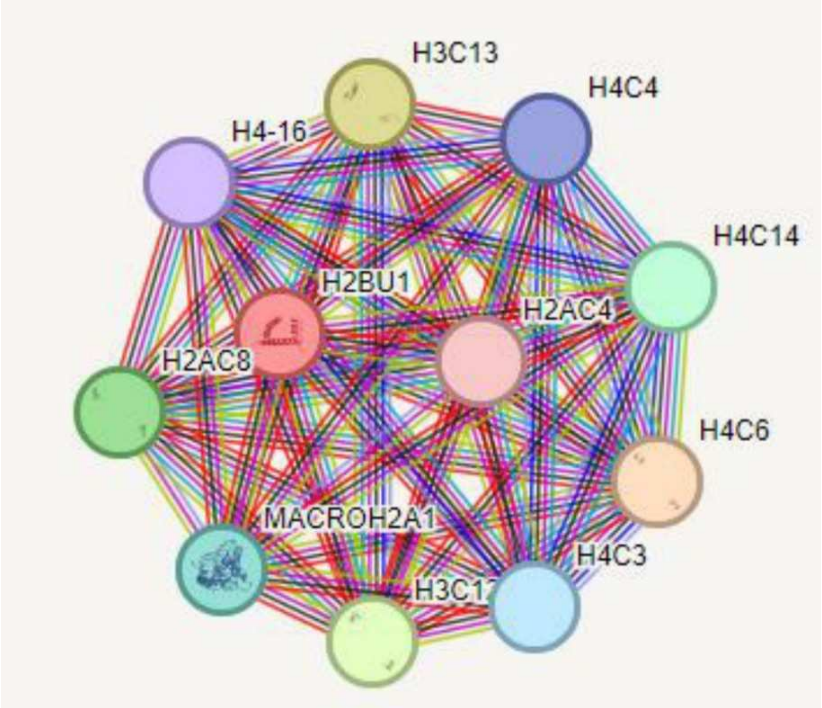

Figure S1D: String network analysis of proteins interacting with FTL.
String network analysis using STRING interactive website (https://string-db.org/cgi/input?sessionId=bZa9VJumLnb8&input_page_show_search=on) using basic settings: full STRING network and evidence = true. Colored nodes represent query proteins, filled nodes: a 3D structure is known or predicted. Bluegreen edges: protein-protein associations from curated databases, magenta edges: protein-protein associations from experimentally determined results, green edges: predicted protein-protein associations from gene neighborhoods, red edges: predicted protein-protein associations from gene fusions, blue edges: predicted protein-protein associations from gene co-occurrences, light green edges: predicted protein-protein associations from text mining, black edges: predicted protein-protein associations from gene co-expression, light blue edges: predicted protein-protein associations from protein homology. Colors of nodes refer to the same characteristics as described above for edges.

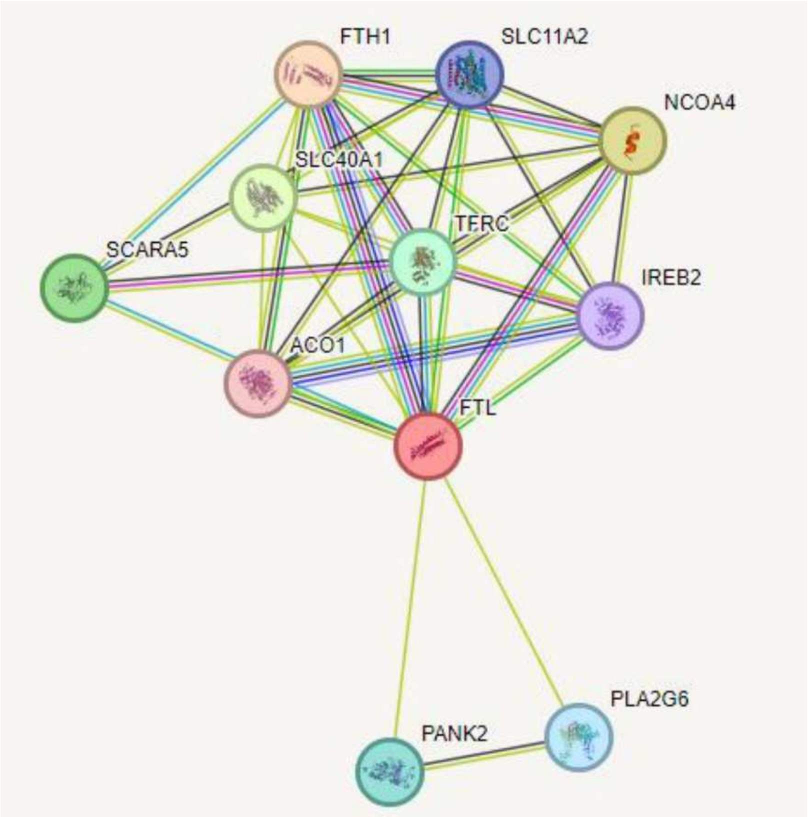

Figure S1E: String network analysis of proteins interacting with MX1.
String network analysis using STRING interactive website (https://string-db.org/cgi/input?sessionId=bZa9VJumLnb8&input_page_show_search=on) using basic settings: full STRING network and evidence = true. Colored nodes represent query proteins, filled nodes: a 3D structure is known or predicted. Bluegreen edges: protein-protein associations from curated databases, magenta edges: protein-protein associations from experimentally determined results, green edges: predicted protein-protein associations from gene neighborhoods, red edges: predicted protein-protein associations from gene fusions, blue edges: predicted protein-protein associations from gene co-occurrences, light green edges: predicted protein-protein associations from text mining, black edges: predicted protein-protein associations from gene co-expression, light blue edges: predicted protein-protein associations from protein homology. Colors of nodes refer to the same characteristics as described above for edges.

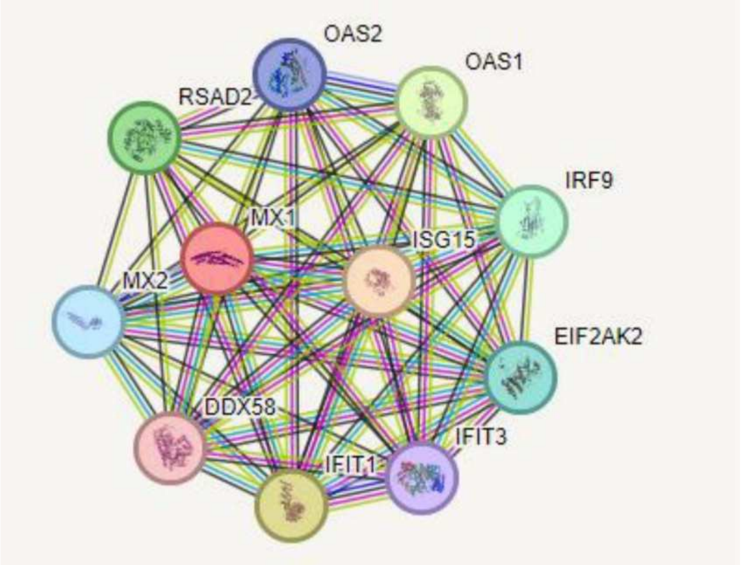

Figure S1F: String network analysis of proteins interacting with C1QC.

String network analysis using STRING interactive website (https://string-db.org/cgi/input?sessionId=bZa9VJumLnb8&input_page_show_search=on) using basic settings: full STRING network and evidence = true. Colored nodes represent query proteins, filled nodes: a 3D structure is known or predicted. Bluegreen edges: protein-protein associations from curated databases, magenta edges: protein-protein associations from experimentally determined results, green edges: predicted protein-protein associations from gene neighborhoods, red edges: predicted protein-protein associations from gene fusions, blue edges: predicted protein-protein associations from gene co-occurrences, light green edges: predicted protein-protein associations from text mining, black edges: predicted protein-protein associations from gene co-expression, light blue edges: predicted protein-protein associations from protein homology. Colors of nodes refer to the same characteristics as described above for edges.

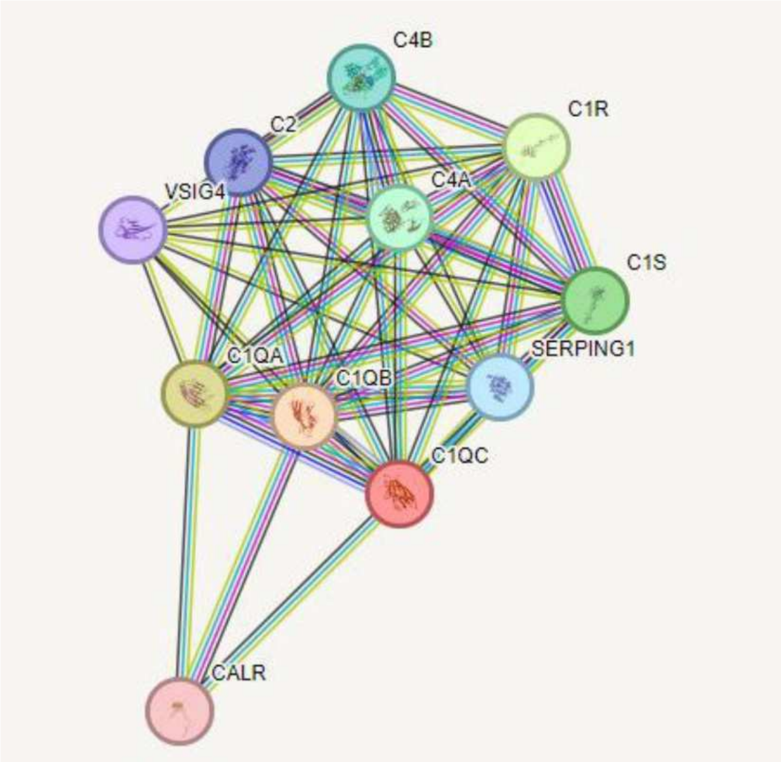

Figure S1G: String network analysis of proteins interacting with HAMP.

String network analysis using STRING interactive website (https://string-db.org/cgi/input?sessionId=bZa9VJumLnb8&input_page_show_search=on) using basic settings: full STRING network and evidence = true. Colored nodes represent query proteins, filled nodes: a 3D structure is known or predicted. Bluegreen edges: protein-protein associations from curated databases, magenta edges: protein-protein associations from experimentally determined results, green edges: predicted protein-protein associations from gene neighborhoods, red edges: predicted protein-protein associations from gene fusions, blue edges: predicted protein-protein associations from gene co-occurrences, light green edges: predicted protein-protein associations from text mining, black edges: predicted protein-protein associations from gene co-expression, light blue edges: predicted protein-protein associations from protein homology. Colors of nodes refer to the same characteristics as described above for edges.

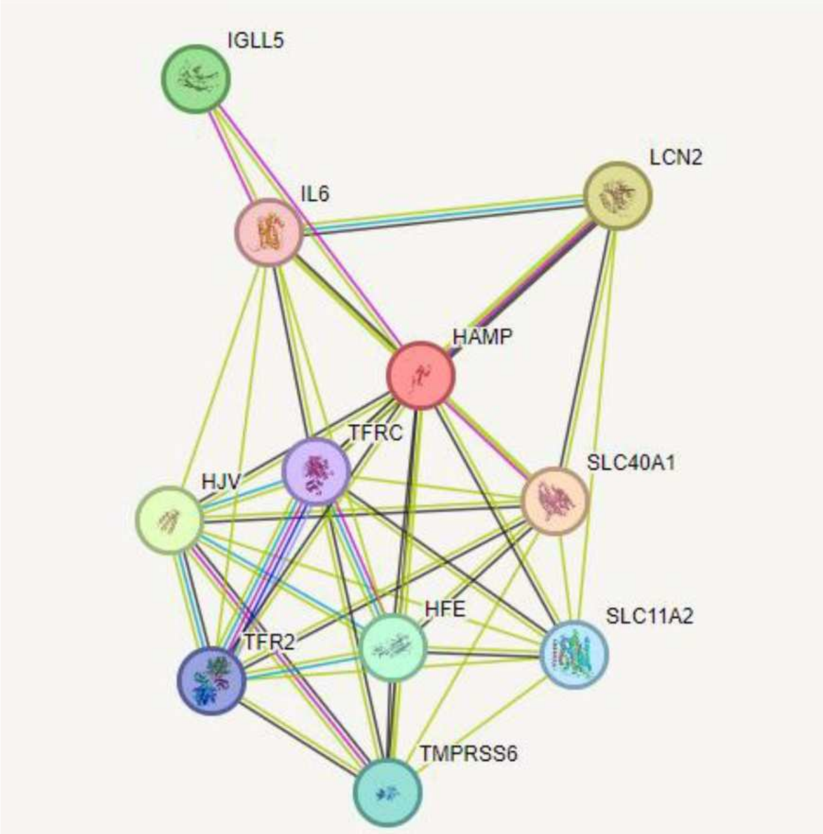

Figure S2A: PCA from proteome LC-MS data.
Principal Component Analysis (PCA) plot for protein expression values of healthy controls (black) and infected participants (red).

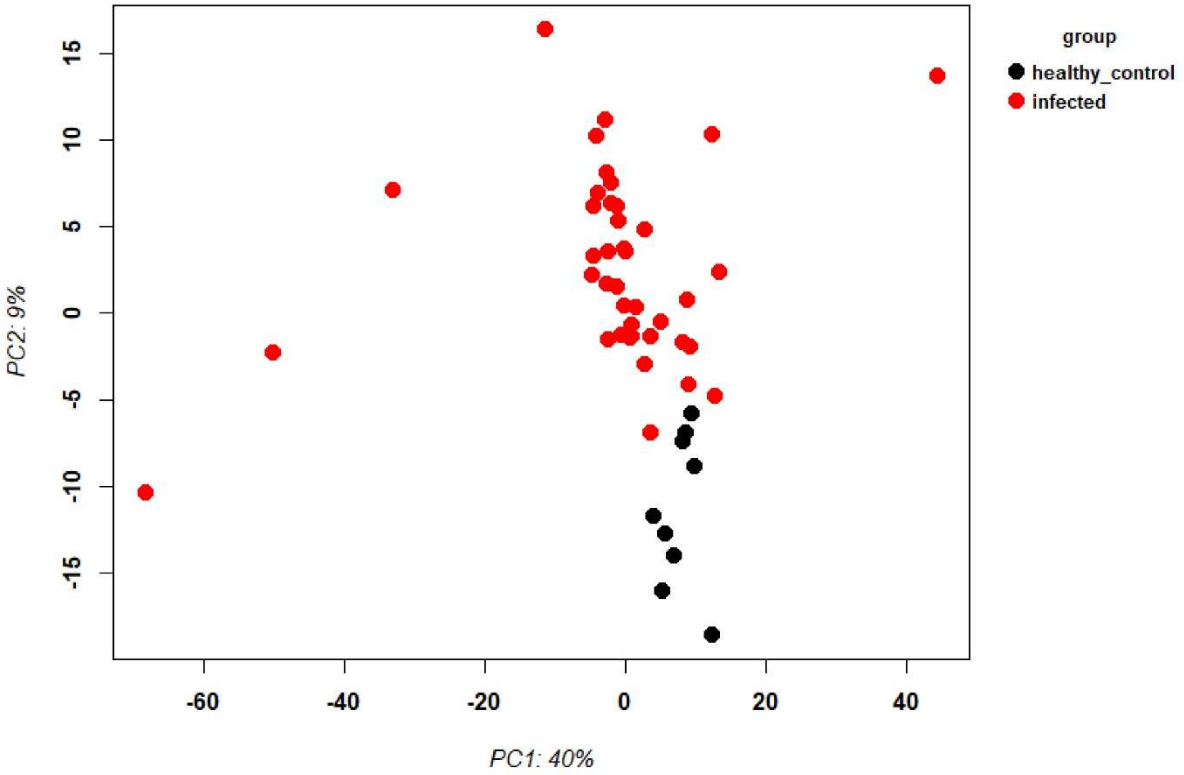

Figure S2B: Summary of DEPs from contrasts of LC-MS data.
Bar plot representing numbers of up- and down-regulated DEPs for the individual contrasts.

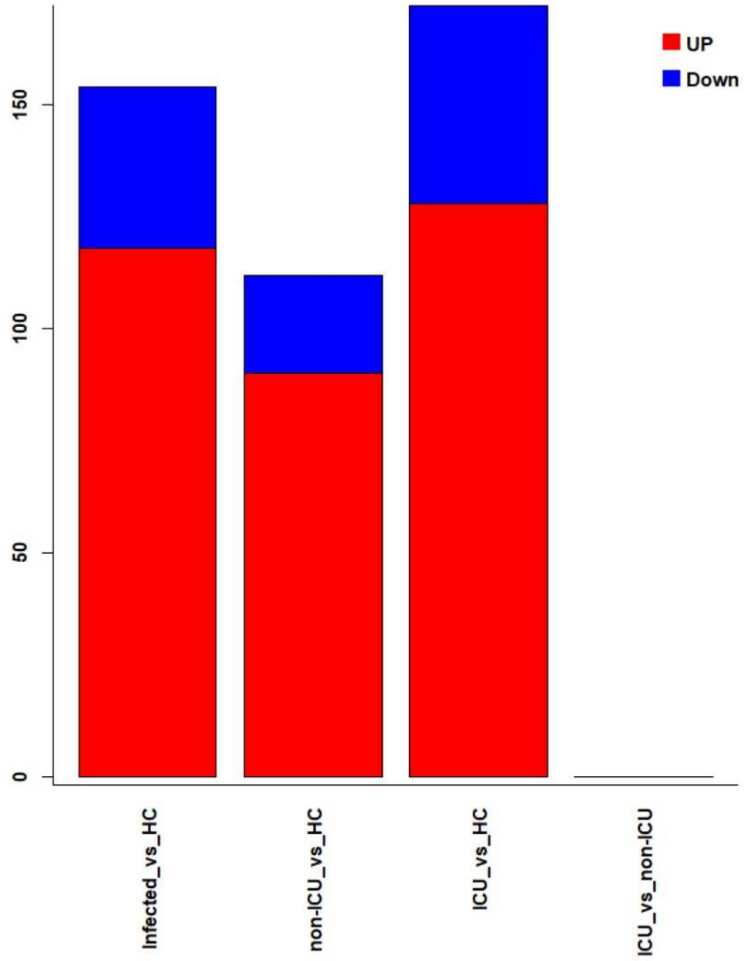

Figure S2C: Volcano plot of contrasts using LC-MS data.

Volcano plot of DEPs for infected patients versus healthy controls. 'Upregulated': higher in ICU, 'down-regulated': higher in non-ICU patients. Y-axis: $-\log_{10}$ BH multiple testing adjusted p-values, x-axis: $\log_2$ fold change. DEPs are colored red, the top 20 up- and downregulated (by log-fold change) DEPs are labeled. Blue: not significant proteins with an adjusted $P < 0.05$. Yellow: not significant proteins with an absolute $\log_2$ change > 0.58.

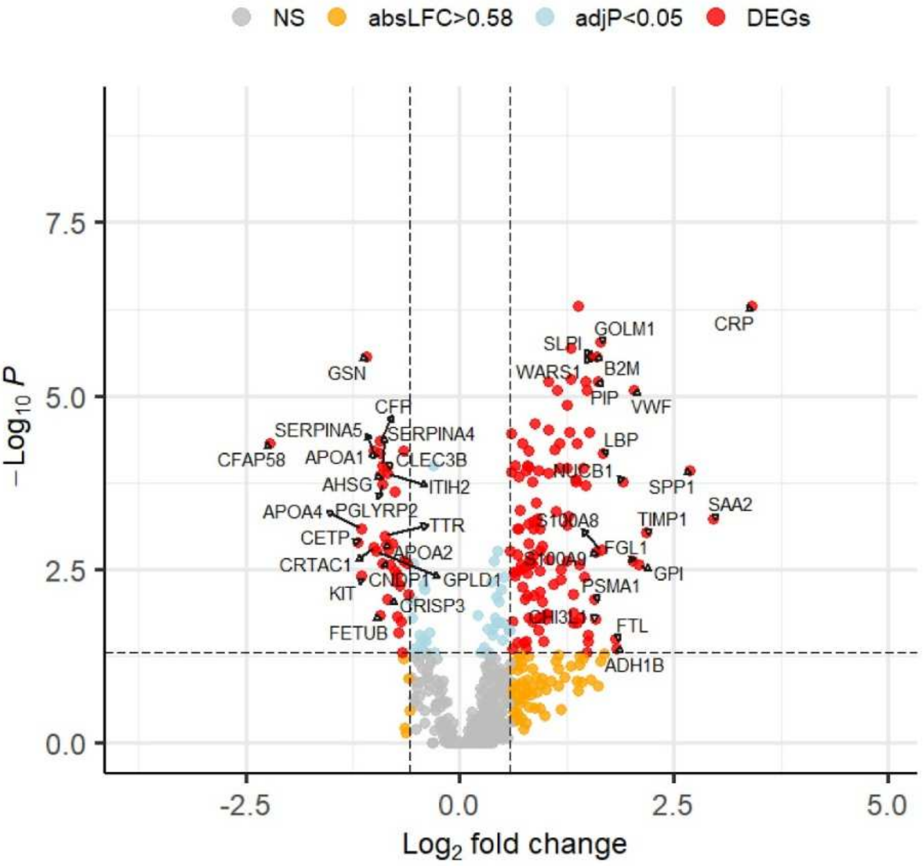

Figure S2D: Scatter plots comparing expression levels for selected DEPs from SOMAscan and LC-MS analyses.

Scatter plots of DEPs from selected proteins. Dots represent log_2-fold differences of infected patients versus healthy controls for SOMAscan (Y-axis) and LC-MS analyses (X-axis).

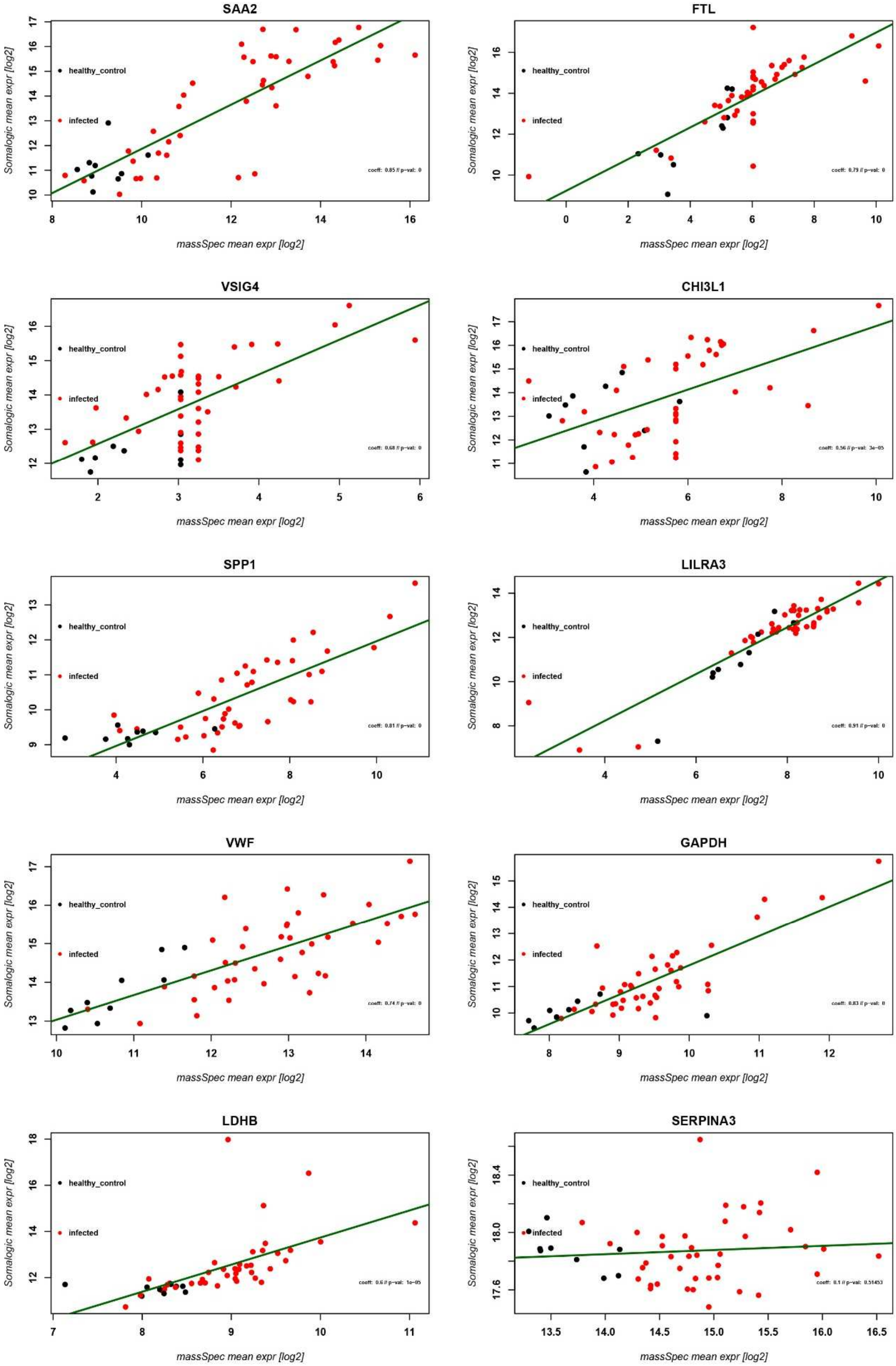

Figure S3: Volcano plot of contrasts old versus young.
Volcano plot of DEPs for infected patients versus healthy controls. ‘Upregulated’: higher in OLD, ‘down-regulated’: higher in young patients. Y-axis: -$\log_{10}$ BH multiple testing adjusted p-values, x-axis: $\log_2$ fold change. DEPs are colored red, the top 20 up- and downregulated (by log-fold change) DEPs are labeled. Blue: not significant proteins with an adjusted P < 0.05. Yellow: not significant proteins with an absolute $\log_2$ change > 0.58.

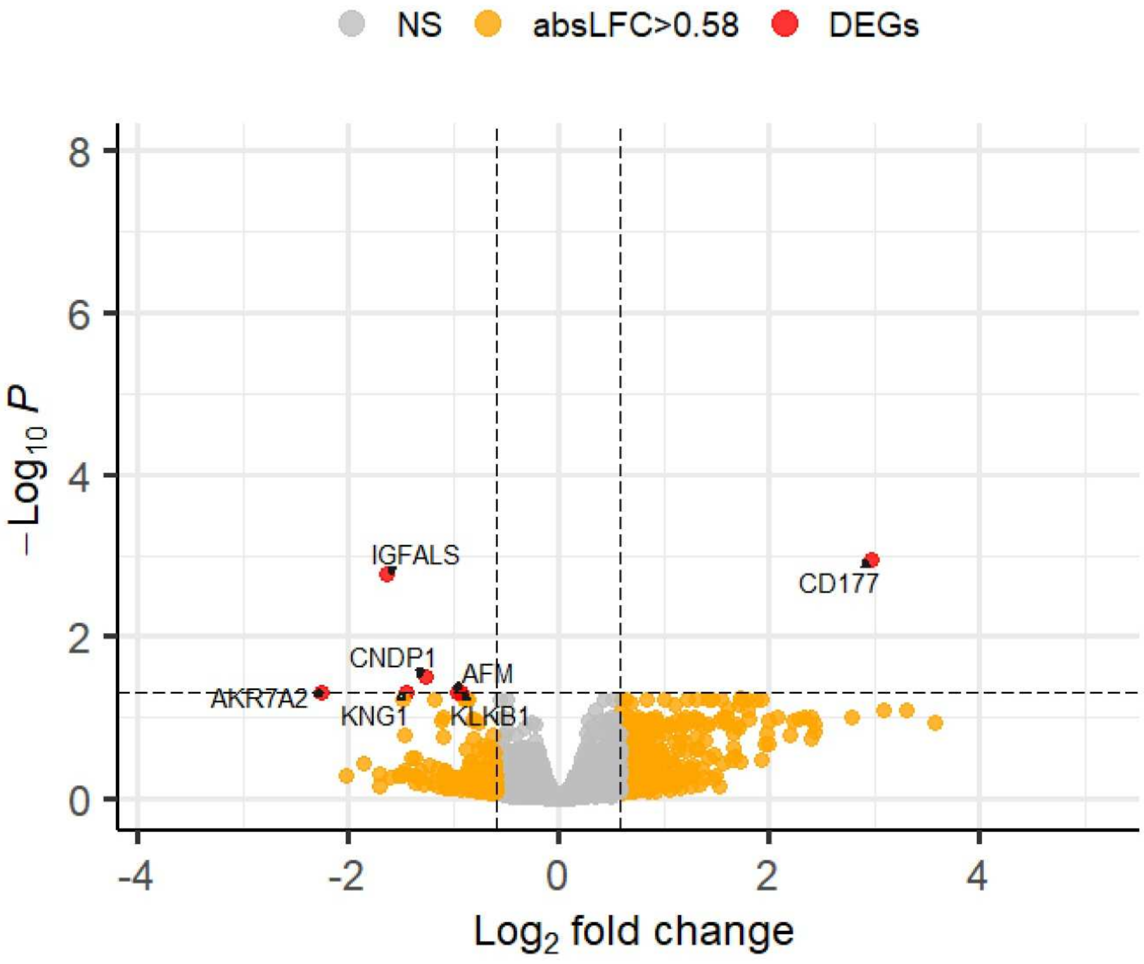

Figure S4A: Summary of DEPs from contrasts of SOMAscan data.
Bar plot representing numbers of up- and down-regulated DEPs for the individual contrasts.

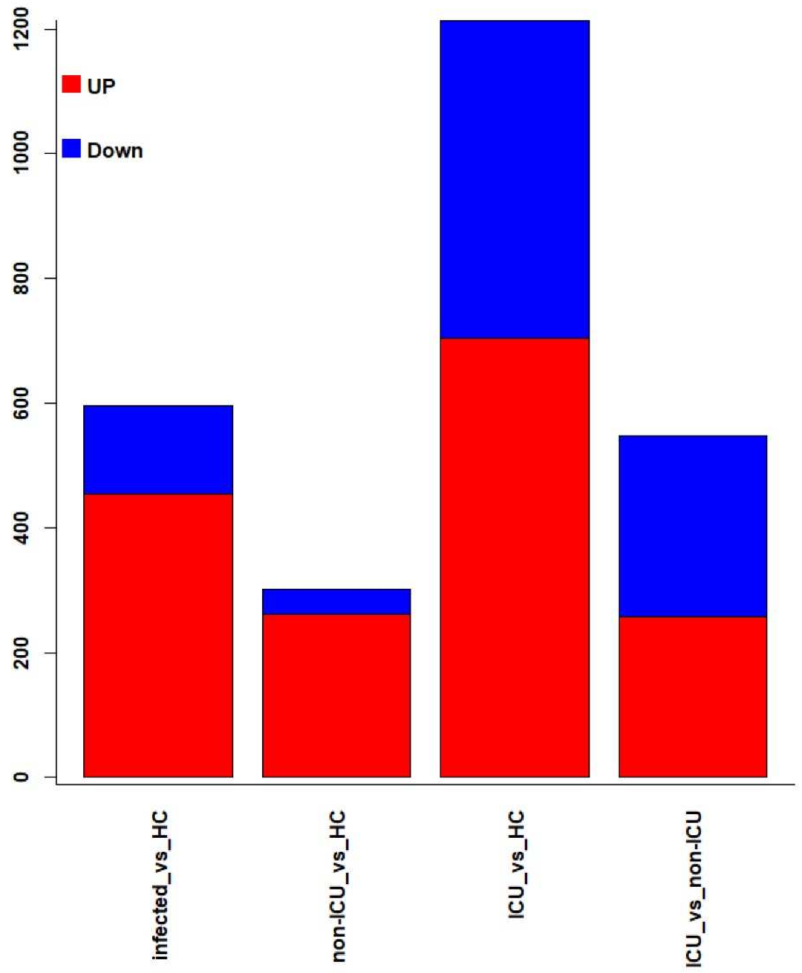

Figure S4B: Pathways for DEPs from comparison of non-ICU patients versus healthy controls (all DEPs).

Functional analysis of all regulated DEPs. Bars represent top 10 (by p-value) pathway hits of EnrichR analysis (Y-axis for gene ratios and color for p-values) from databases "Reactome_2022", "GO_Biological_Process_2025", and "KEGG_2021_Human" (from left to right).

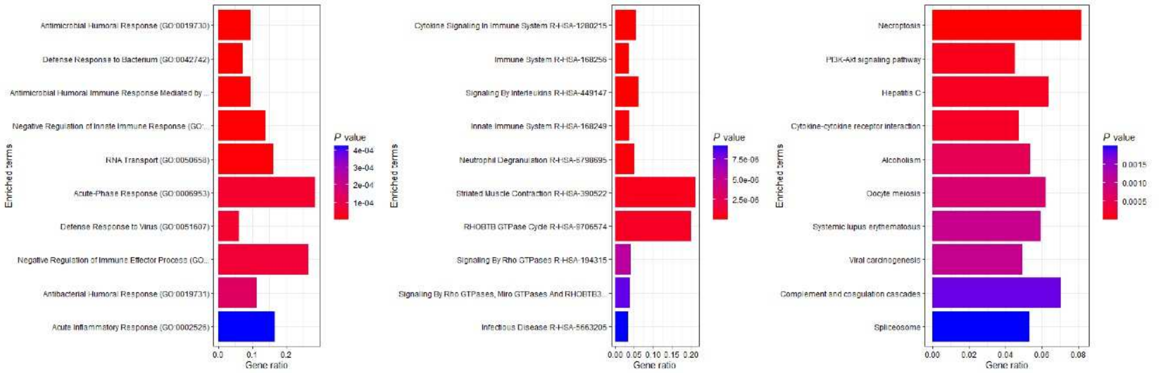

Figure S4C: Pathways for DEPs from comparison of ICU patients versus Healthy controls (all DEPs).

Functional analysis of all regulated DEPs. Bars represent top 10 (by p-value) pathway hits of EnrichR analysis (Y-axis for gene ratios and color for p-values) from databases "Reactome_2022", "GO_Biological_Process_2025", and "KEGG_2021_Human" (from left to right).

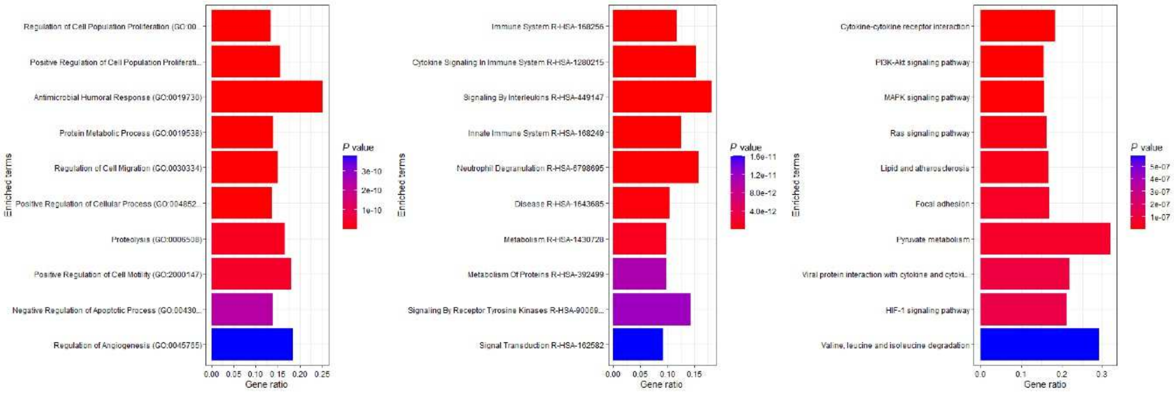

Figure S4D: VENN diagrams of overlaps for ICU patients and no-ICU patients versus healthy controls.

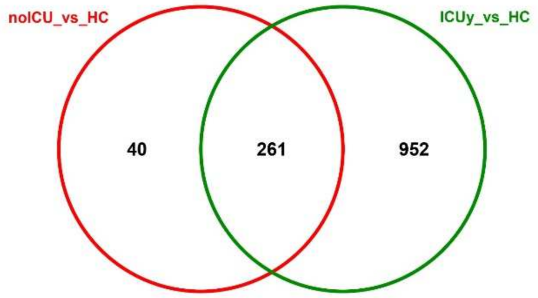

Figure S4E: Pathways for overlapping 261 genes in ICU patients and no-ICU patients versus healthy controls.
Functional analysis of overlapping DEPs. Bars represent top 10 (by p-value) pathway hits of EnrichR analysis (Y-axis for gene ratios and color for p-values) from databases "Reactome_2022", "GO_Biological_Process_2025", and "KEGG_2021_Human" (from left to right).

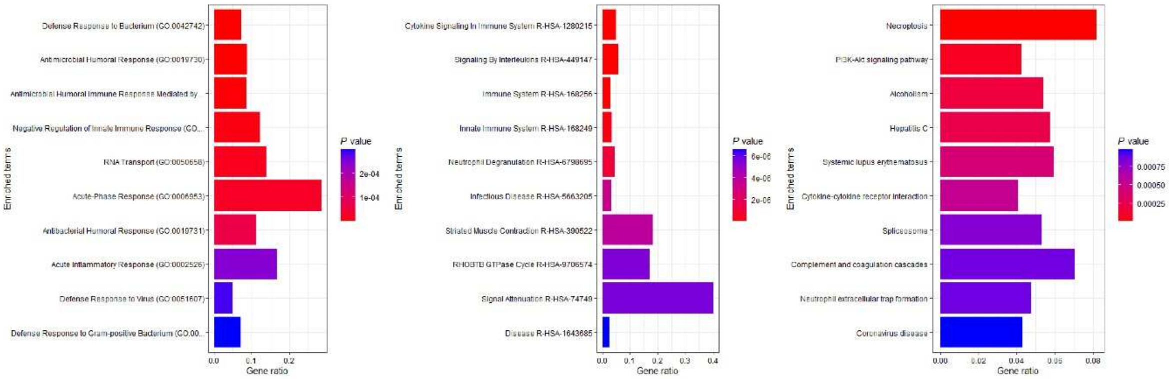

Figure S5A: Overlap with external influenza studies mild cases versus healthy controls.
Bar plots presenting log_2 changes from contrast for selected proteins. Red: DEPs significant for this contrast.

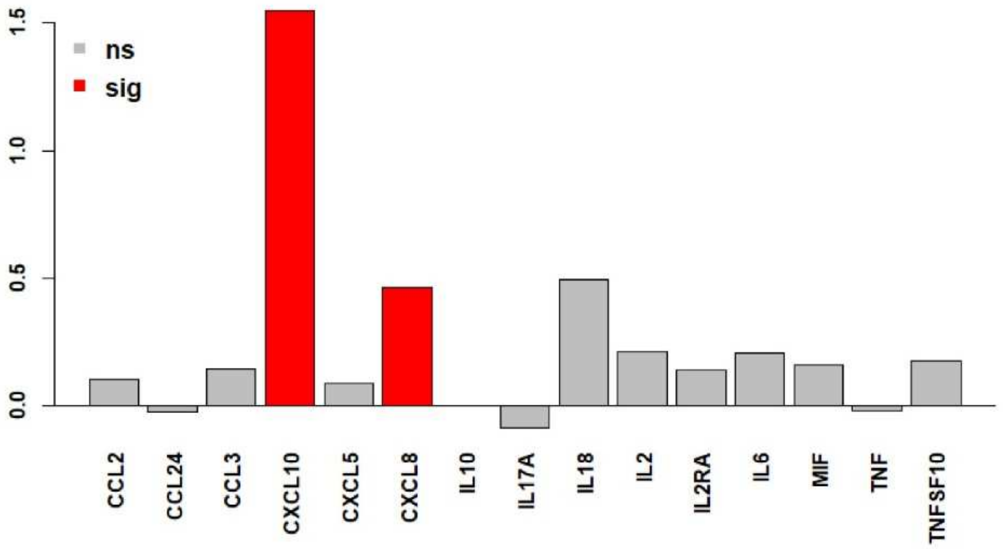

Figure S5B: Overlap external influenza studies ICU cases versus healthy controls.
Bar plots presenting log_2 changes from contrast for selected proteins. Red: DEPs significant for this contrast.

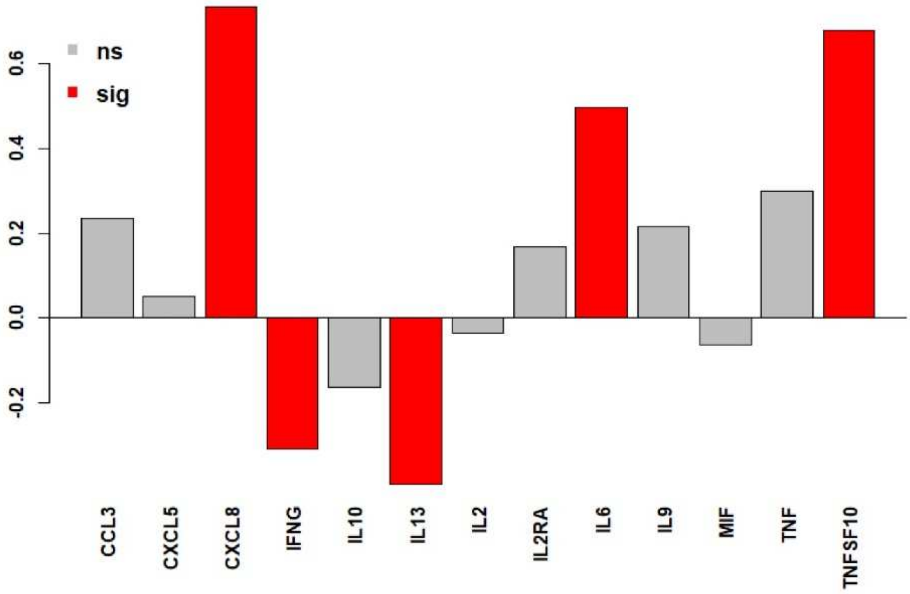

Figure S5C: Overlap external influenza studies ICU versus no-ICU cases.
Bar plots presenting $\log_2$ changes from contrast for selected proteins. Red: DEPs significant for this contrast.

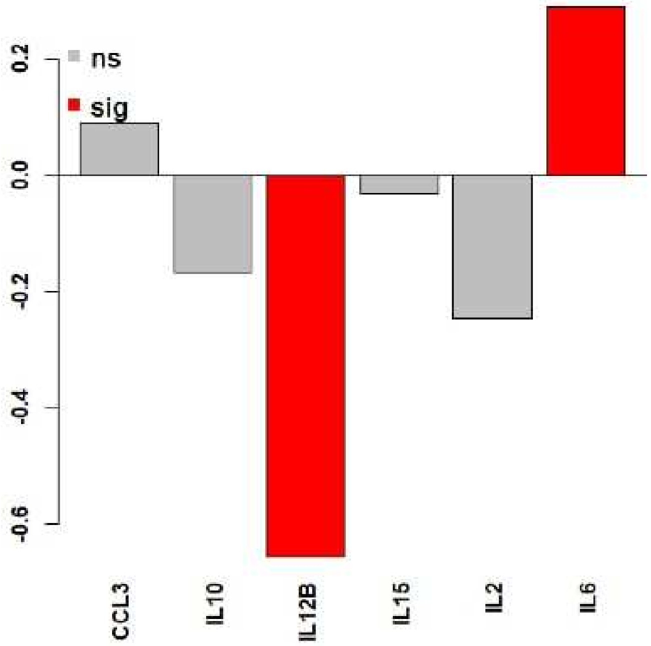

Figure S6: Expression of SAA1 and SAA2.
Box plot of the SAA1 and SAA2 expression levels for healthy controls (HC_ICU_n), infected non-ICU and ICU patients. Box represents first quartile, median, and third quartile; whiskers represent values within 1.5 × IQR (interquartile range, Q3–Q1).

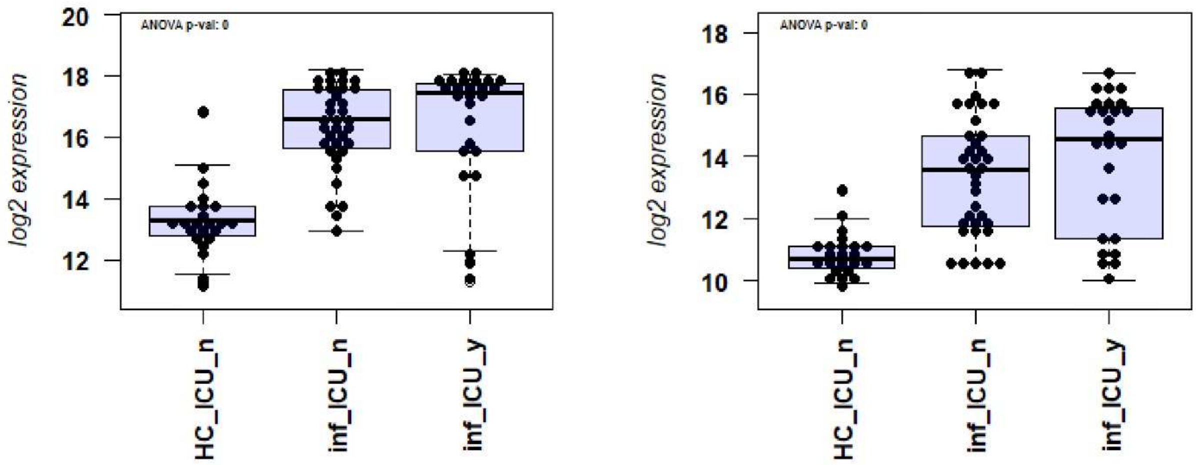

Supplement: Supplementary file 1 [file DataSheet1.pdf]
