# Supplementary material for: Analysis of blood proteome in influenza-infected patients reveals new insights into the host response signatures distinguishing mild severe infections
Source: Front Immunol. 2025 Nov 19;16:1693728. doi: 10.3389/fimmu.2025.1693728 (PMC12672548; doi:10.3389/fimmu.2025.1693728)
Supplement: Supplementary file 3 [file Table3.docx]

#### Table_S5_DEP_functions_070725

| **Protein symbol** | **Identified as ..** | **Gene name** | **Known function** |
| --- | --- | --- | --- |
| SAA1 | DEP UP in INF versus HC | Serum Amyloid A1 | Enables G protein-coupled receptor binding activity. Involved in several processes, including leukocyte chemotaxis; positive regulation of cytokine production; and positive regulation of cytosolic calcium ion concentration. Located in extracellular exosome. Part of cytoplasmic microtubule. |
| H2BU1 | DEP UP in INF versus HC | H2B Clustered Histone 26 | No record in alliance database |
| SAA2 | DEP UP in INF versus HC | Serum Amyloid A2 | Predicted to be involved in response to stilbenoid. Located in extracellular exosome. |
| H2BC12 | DEP UP in INF versus HC | H2B Clustered Histone 12 | Predicted to enable DNA binding activity and protein heterodimerization activity. Predicted to be a structural constituent of chromatin. Involved in antimicrobial humoral immune response mediated by antimicrobial peptide and innate immune response in mucosa. Located in cytosol; extracellular space; and nucleoplasm. |
| H2AC1 | DEP UP in INF versus HC | H2A Clustered Histone 1 | Predicted to enable DNA binding activity. Predicted to act upstream of or within chromatin organization. Located in chromosome, telomeric region; extracellular exosome; and nucleus. |
| H2AW | DEP UP in INF versus HC | H2A Clustered Histone 25 | No record in alliance database |
| H2BC21 | DEP UP in INF versus HC | H2B Clustered Histone 21 | Predicted to enable DNA binding activity and protein heterodimerization activity and a structural constituent of chromatin, involved in antimicrobial humoral immune response mediated by antimicrobial peptide and innate immune response in mucosa. |
| FTL | DEP UP in INF versus HC | Ferritin Light Chain | Enables identical protein binding activity and iron ion binding activity. Predicted to be involved in intracellular sequestering of iron ion. Located in autolysosome. Part of intracellular ferritin complex. Implicated in basal ganglia disease; hyperferritinemia-cataract syndrome; neurodegeneration with brain iron accumulation 3; and neurodegenerative disease. Biomarker of COVID-19. |
| FTH1 | DEP UP in INF versus HC | Ferritin Heavy Chain 1 | Enables identical protein binding activity and iron ion sequestering activity. Involved in negative regulation of fibroblast proliferation. Located in autolysosome. Implicated in hemochromatosis type 5. Biomarker of COVID-19. |
| MX1 | DEP UP in INF versus HC | MX Dynamin Like GTPase 1 | Enables identical protein binding activity. Involved in interleukin-27-mediated signaling pathway; negative regulation of viral genome replication; and response to virus. Located in cytosol; nuclear membrane; and perinuclear region of cytoplasm. Implicated in alopecia areata; avian influenza; liver disease; and prostate cancer. Biomarker of Human papillomavirus infectious disease. |
| PZP | DEP DOWN in INF versus HC | PZP Alpha-2-Macroglobulin Like | Predicted to enable endopeptidase inhibitor activity and protease binding activity, involved in female pregnancy. Located in blood microparticle and extracellular exosome. Colocalizes with collagen-containing extracellular matrix. |
| C1orf162 | DEP DOWN in INF versus HC | Chromosome 1 Open Reading Frame 162 | Predicted to be located in membrane. |
| COL11A2 | DEP DOWN in INF versus HC | Collagen Type XI Alpha 2 Chain | An extracellular matrix structural constituent conferring tensile strength. Involved in cartilage development; collagen fibril organization; and soft palate development. Acts upstream of or within sensory perception of sound. Located in collagen-containing extracellular matrix. Implicated in cleft palate; nonsyndromic deafness (multiple); and osteochondrodysplasia (multiple). |
| ANTXR2 | DEP DOWN in INF versus HC | ANTXR Cell Adhesion Molecule 2 | Predicted to enable transmembrane signaling receptor activity. Predicted to act upstream of or within reproductive process. Predicted to be located in external side of plasma membrane. Predicted to be active in cell surface and plasma membrane. Implicated in fibroma and hyaline fibromatosis syndrome. |
| CILP2 | DEP DOWN in INF versus HC | Cartilage Intermediate Layer Protein 2 | Located in extracellular exosome. |
| MDGA2 | DEP DOWN in INF versus HC | MAM Domain Containing Glycosylphosphatidylinositol Anchor 2 | Predicted to be involved in regulation of presynapse assembly; regulation of synaptic membrane adhesion; and spinal cord motor neuron differentiation. Predicted to act upstream of or within neuron migration and pattern specification process. Predicted to be located in extracellular region and plasma membrane. Predicted to be active in GABA-ergic synapse and glutamatergic synapse. |
| CHAD | DEP DOWN in INF versus HC | Chondroadherin | Predicted to act upstream of or within bone development and negative regulation of bone trabecula formation. Predicted to be located in extracellular region. Predicted to be active in extracellular matrix and extracellular space. |
| PF4 | DEP DOWN in INF versus HC | Platelet Factor 4 | Enables CXCR3 chemokine receptor binding activity and heparin binding activity. Involved in several processes, including negative regulation of extrinsic apoptotic signaling pathway in absence of ligand; regulation of macromolecule biosynthetic process; and regulation of myeloid cell differentiation. Located in cytoplasm. Implicated in multiple myeloma. Biomarker of systemic scleroderma. |
| BDNF | DEP DOWN in INF versus HC | Brain Derived Neurotrophic Factor | Predicted to enable growth factor activity and nerve growth factor receptor binding activity. Involved in nervous system development and positive regulation of nervous system development. Predicted to be located in perinuclear region of cytoplasm and secretory granule. Predicted to be active in several cellular components, including glutamatergic synapse; hippocampal mossy fiber to CA3 synapse; and secretory vesicle. Implicated in several diseases, including cognitive disorder (multiple); congenital central hypoventilation syndrome; neurodegenerative disease (multiple); obstructive sleep apnea; and status epilepticus. Biomarker of several diseases, including autoimmune disease (multiple); depressive disorder (multiple); lung disease (multiple); neurodegenerative disease (multiple); and open-angle glaucoma (multiple). |
| HPGDS | DEP DOWN in INF versus HC | Hematopoietic Prostaglandin D Synthase | Predicted to enable metal ion binding activity, prostaglandin-D synthase activity, and protein homodimerization activity. Involved in prostaglandin metabolic process. |
| H2BC12 | DEP UP in ICU versus non-ICU patients | H2B Clustered Histone 12 | Predicted to enable DNA binding activity and protein heterodimerization activity. Predicted to be a structural constituent of chromatin. Involved in antimicrobial humoral immune response mediated by antimicrobial peptide and innate immune response in mucosa. Located in cytosol; extracellular space; and nucleoplasm. |
| NTN1 | DEP UP in ICU versus non-ICU patients | Netrin 1 | Predicted to enable DNA-binding transcription factor activity, RNA polymerase II-specific and RNA polymerase II cis-regulatory region sequence-specific DNA binding activity. Predicted to be involved in several processes, including Cdc42 protein signal transduction; plasma membrane bounded cell projection organization; and positive regulation of axon extension. Predicted to act upstream of or within several processes, including generation of neurons; inner ear morphogenesis; and regulation of neurogenesis. Located in several cellular components, including actin cytoskeleton; cytosol; and nucleoplasm. Implicated in congenital mirror movement disorder. |
| H2BU1 | DEP UP in ICU versus non-ICU patients | H2B Clustered Histone 26 | No record in alliance database |
| H2AC1 | DEP UP in ICU versus non-ICU patients | H2A Clustered Histone 1 | Predicted to enable DNA binding activity. Predicted to act upstream of or within chromatin organization. Located in chromosome, telomeric region; extracellular exosome; and nucleus. |
| FGFBP1 | DEP UP in ICU versus non-ICU patients | Fibroblast Growth Factor Binding Protein 1 | Predicted to enable growth factor binding activity. Involved in positive regulation of blood vessel endothelial cell proliferation involved in sprouting angiogenesis and positive regulation of cell migration involved in sprouting angiogenesis. Predicted to be located in extracellular space. |
| SFRP5 | DEP UP in ICU versus non-ICU patients | Secreted Frizzled Related Protein 5 | Predicted to enable Wnt-protein binding activity. Involved in negative regulation of cell population proliferation and negative regulation of signal transduction. Predicted to be located in extracellular region. Predicted to be active in extracellular space. |
| IL1RL1 | DEP UP in ICU versus non-ICU patients | Interleukin 1 Receptor Like 1 | Enables interleukin-33 receptor activity. Involved in interleukin-33-mediated signaling pathway. Located in cytosol; focal adhesion; and plasma membrane. Implicated in asthma and coronary artery disease. Biomarker of several diseases, including Human papillomavirus infectious disease; hemolytic-uremic syndrome; lung disease (multiple); paracoccidioidomycosis; and tongue squamous cell carcinoma. |
| CELF2 | DEP UP in ICU versus non-ICU patients | CUGBP Elav-Like Family Member 2 | Enables pre-mRNA binding activity. Involved in mRNA splice site recognition. Located in Flemming body and nucleoplasm. Implicated in developmental and epileptic encephalopathy 97. |
| H2AW |  | H2A Clustered Histone 25 | No record in alliance database |
| MDK | DEP UP in ICU versus non-ICU patients | Midkine | Enables heparan sulfate binding activity and heparin binding activity. Involved in several processes, including negative regulation of apoptotic process; positive regulation of cell migration; and regulation of leukocyte cell-cell adhesion. Colocalizes with collagen-containing extracellular matrix. Implicated in brain ischemia. Biomarker of endometriosis. |
| C1orf162 | DEP DOWN in ICU versus non-ICU patients | Chromosome 1 Open Reading Frame 162 | Predicted to be located in membrane. |
| LRRC15 | DEP DOWN in ICU versus non-ICU patients | Leucine Rich Repeat Containing 15 | Enables several functions, including fibronectin binding activity; laminin binding activity; and protein sequestering activity. Involved in several processes, including negative regulation of protein localization to plasma membrane; negative regulation of viral entry into host cell; and receptor-mediated virion attachment to host cell. Located in plasma membrane. Is active in apical plasma membrane. Colocalizes with collagen-containing extracellular matrix. |
| DNM1L | DEP DOWN in ICU versus non-ICU patients | Dynamin 1 Like | Enables several functions, including GTP-dependent protein binding activity; enzyme binding activity; and protein homodimerization activity. Involved in several processes, including mitochondrion organization; peroxisome fission; and regulation of mitochondrion organization. Acts upstream of with a positive effect on protein-containing complex assembly. Acts upstream of or within mitochondrion morphogenesis. Located in several cellular components, including mitochondrial outer membrane; perinuclear region of cytoplasm; and peroxisome. Part of protein-containing complex. Colocalizes with microtubule and mitochondrion-derived vesicle. Implicated in encephalopathy due to defective mitochondrial and peroxisomal fission 1 and optic atrophy 5. Biomarker of Alzheimer's disease and pulmonary fibrosis. |
| TAPBPL | DEP DOWN in ICU versus non-ICU patients | TAP Binding Protein Like | Enables MHC class I protein complex binding activity. Involved in negative regulation of antigen processing and presentation of peptide antigen via MHC class I and peptide antigen assembly with MHC class I protein complex. Located in endoplasmic reticulum. |
| LTO1 | DEP DOWN in ICU versus non-ICU patients | LTO1 Maturation Factor Of ABCE1 | Involved in protein maturation by [4Fe-4S] cluster transfer; ribosomal large subunit biogenesis; and translational initiation. Predicted to be located in nucleus. |
| TCF12 | DEP DOWN in ICU versus non-ICU patients | Transcription Factor 12 | Enables several functions, including DNA-binding transcription activator activity, RNA polymerase II-specific; SMAD binding activity; and bHLH transcription factor binding activity. Contributes to transcription cis-regulatory region binding activity. Involved in positive regulation of transcription by RNA polymerase II and response to gonadotropin-releasing hormone. Located in cytoplasm and nuclear speck. Part of RNA polymerase II transcription regulator complex and chromatin. Implicated in craniosynostosis and hypogonadotropic hypogonadism. |
| TP53I11 | DEP DOWN in ICU versus non-ICU patients | Tumor Protein P53 Inducible Protein 11 | Predicted to be involved in negative regulation of cell population proliferation. Predicted to be located in membrane. |
| CLEC4C | DEP DOWN in ICU versus non-ICU patients | C-Type Lectin Domain Family 4 Member C | Predicted to enable carbohydrate binding activity. Predicted to be involved in antifungal innate immune response. Predicted to act upstream of or within positive regulation of release of sequestered calcium ion into cytosol. Predicted to be located in ficolin-1-rich granule membrane; plasma membrane; and tertiary granule membrane. Predicted to be active in external side of plasma membrane. |
| IL36RN | DEP DOWN in ICU versus non-ICU patients | Interleukin 36 Receptor Antagonist | Predicted to enable interleukin-1 receptor antagonist activity. Involved in antifungal humoral response; negative regulation of cytokine production; and negative regulation of cytokine-mediated signaling pathway. Predicted to be located in extracellular region. Predicted to be active in extracellular space. Implicated in pustular psoriasis 14. |
| ASNS | DEP DOWN in ICU versus non-ICU patients | Asparagine Synthetase (Glutamine-Hydrolyzing) | Predicted to enable asparagine synthase (glutamine-hydrolyzing) activity, involved in cellular response to glucose starvation; negative regulation of apoptotic process; and positive regulation of mitotic cell cycle. |

List of top DEPs identified in different contrasts for which functions are known. Information on gene symbols and names from Gene Cards (GeneCards), information on gene functions from (Alliance_of_Genome_Resources). Note that the description of functions are exact citations/wordings from the Alliance of Genome Database.

**References**

Alliance_of_Genome_Resources Alliance of Genome Resources. <https://www.alliancegenome.org/>.
