# Supplementary material for: Analysis of blood proteome in influenza-infected patients reveals new insights into the host response signatures distinguishing mild severe infections
Source: Front Immunol. 2025 Nov 19;16:1693728. doi: 10.3389/fimmu.2025.1693728 (PMC12672548; doi:10.3389/fimmu.2025.1693728)
Supplement: Supplementary file 9 [file Table8.docx]

**Table S8: Number DEGs proteome and transcriptome**

| **Contrast** | **Up-regulated** | **Down-regulated** | **Total** |
| --- | --- | --- | --- |
| Proteome ICU vs healthy controls | 590 | 402 | 992 |
| Proteome non-ICU vs healthy controls | 265 | 42 | 307 |
| Proteome ICU vs non-ICU | 134 | 188 | 322 |
| Transcriptome ICU vs healthy controls | 962 | 739 | 1701 |
| Transcriptome non-ICU vs healthy controls | 557 | 313 | 870 |
| Transcriptome ICU vs non-ICU | 73 | 28 | 101 |
